# Promotion of Water as Solvent in Amination of 4-Chloropyrrolopyrimidines and Related Heterocycles under Acidic Conditions

**DOI:** 10.1021/acsomega.3c09673

**Published:** 2024-03-12

**Authors:** Shuhei Yasuda, Hanne Svergja, Cecilie Elisabeth Olsen, Bård Helge Hoff

**Affiliations:** Department of Chemistry, Norwegian University of Science and Technology (NTNU), Ho̷gskoleringen 5, NO-7491 Trondheim, Norway

## Abstract

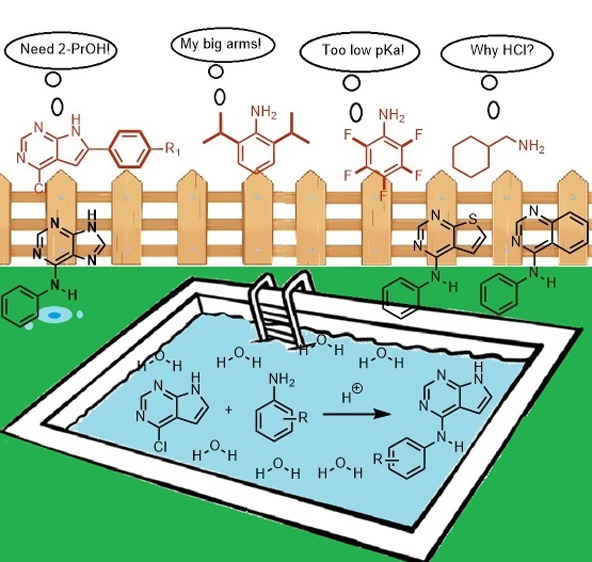

A switch of reaction medium from organic solvents to water can improve the safety and lower the cost of production processes. Hydrochloric acid-promoted amination of fused pyrimidines has been studied using 4-chloro-7*H*-pyrrolo[2,3-*d*]pyrimidine and aniline as model compounds. Higher rate was observed in water than in four alcoholic solvents and DMF. An important aspect is that the amount of acid should be kept low to minimize the competing solvolysis. The substrate scope for the amination in water was evaluated by reacting 4-chloro-7*H*-pyrrolo[2,3-*d]*pyrimidine with 20 aniline derivatives with variance in steric and electronic properties. Preparative useful reactions were seen for 14 of the 20 derivatives. Unsuited nucleophiles are *ortho*-substituted anilines with a p*K*_a_ below 1. Amination of the corresponding quinazoline, thienopyrimidine, and purine also proceeded well in water. Highly lipophilic and crystalline compounds are more efficiently aminated in 2-propanol. Aliphatic and benzylic amines react poorly under acidic conditions, but these aminations can be done in water without acid.

## 1 Introduction

**Figure 1:**
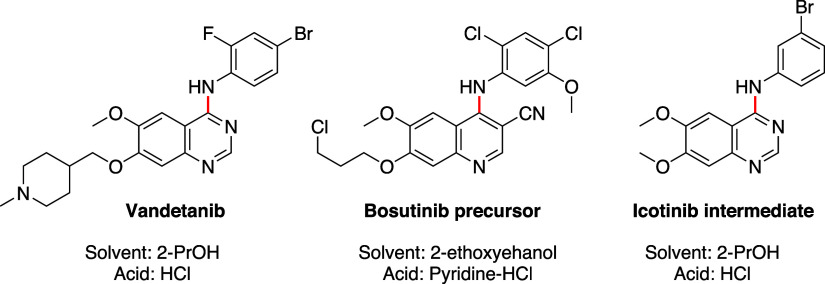
Acid-assisted amination to medically important compounds: Vandetanib ref (25); Bosutinib precursor ref (26); Icotinib intermediate ref (27).

It is estimated that 85–90% of the waste generated in the pharma industry is from solvents.^1^ Thus, a change from organic reaction medium to water without compromising yield will ensure cost savings and reduce hazards.^2,3^ Nucleophilic aromatic substitution with amines is a frequently used reaction in medicinal chemistry.^4^ Depending on the structure and electronic properties of the coupling partners, the transformations can be conducted in different ways. The use of weakly basic/thermal conditions on electron deficient aryls is suitable for benzyl and alkyl amines, but requires an excess of amine or a cobase to quench the generated acid.^5^ Pyrimidines have also been aminated in water with 1 equiv of amine using potassium fluoride as base.^6^*N*-Arylation of amines on less-activated aromatics can also be initiated by NH-deprotonation with strong bases,^7,8^ but is restricted to substrates with no labile groups, and safety aspects can set limitations. With less-activated aromatics, palladium-catalyzed Buchwald–Hartwig aminations are highly efficient.^9−11^ Challenges include regioselectivity when multiple halides are present, when there is possibility for racemization,^12^ and sometimes strictly water-free conditions are needed.^13^ Further, the use of palladium should be minimized due to cost and the risk of contaminating the final product. Acid-catalyzed amination represent an alternative for aromatic heterocycles, and a number of kinase inhibitor drugs rely on processes including an amination step where acid is either added or generated as a byproduct;^14^ some example structures are shown in Figure 1. For pyrrolopyrimidines, amination have been done using HCl,^15−17^ acetic acid,^18^*p*-toluenesulfonic acid,^19^ silver triflate,^20,21^ InCl_3_,^22,23^ and Zn(NO_2_)_2_.^24^

Although acid-induced amination is frequently employed for anilines, we noticed that the published works are mainly of preparative nature and that more in-depth studies on acid-catalyzed amination on heteroaryls are lacking. Among others, the fact that acid is produced in the reaction, and that excess acid has an inhibiting effect on rate seems to be ignored in many synthetic protocols. Additionally, water has not been evaluated as solvent. Naturally, these acid-catalyzed reactions are a balancing act: how can you activate the aryl halide without deactivating the nucleophile. Herein, we report our study of effect of solvent and acid amount on the amination of fused pyrimidines. By evaluating reactivity of 20 different aniline derivatives in amination with 4-chloro-7*H*-pyrrolo[2,3-*d*]pyrimidine and seven fused pyrimidines in reaction with aniline, we show the substrate scope of this transformation and highlight the benefits and limitations of water as reaction medium.

## 2 Results and Discussion

### 2.1 Initial Reactions

**Scheme 1:**
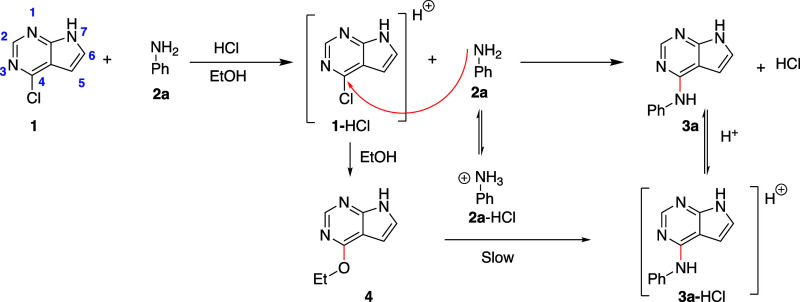
Proposed Intermediates and Equilibriums for the Amination of Pyrrolopyrimidine **1** with **2a** to give **3a** and the Solvolysis Side-Product **4** The numbering system for pyrrolopyrimidines is shown for compound **1**.

Our initial test reactions of acid-catalyzed amination were performed between 4-chloro-7*H*-pyrrolo[2,3-*d*]pyrimidine (**1**), aniline (**2a**) in EtOH using HCl as catalyst, Scheme 1, (see also Supporting Information, Table S1). These studies revealed that the initial rate increased with the amount of HCl, but also that high amount of acid promoted formation of the solvolysis side-product **4**. Thus, the use of 0.1 equiv of acid was seen as a good compromise; the reaction started without a lag time and the formation of the side-product **4** was suppressed. We assume the reaction to proceed as shown in Scheme 1. The pyrrolopyrimidine **1** is not very basic and would be only transiently activated by protonation or hydrogen bonding, lowering the energy barrier for reaction at C-4. The neutral nucleophilic aniline (**2a**) will be in equilibrium with the non-nucleophilic anilinium ion (**2a**-HCl). The position of this equilibrium depends on the amount of acid added at start, the degree of conversion, and the p*K*_a_ of the aniline. The amination generates the product **3a** and one mole equivalent of HCl. The product **3a** is more basic than the starting material and will act as a buffer by forming the corresponding hydrochloride salt (**3a**-HCl). At higher concentration of acid, most of the aniline (**2a**) is inactivated by protonation, which leaves EtOH as a competitive nucleophile giving **4**. Our study indicates that also 4-ethoxy-7*H*-pyrrolo[2,3-*d*]pyrimidine (**4**) slowly converts to the product **3a** (Supporting Information, Figure S1).

### 2.2 Effect of Different Protic Solvents on the Reaction

**Figure 2:**
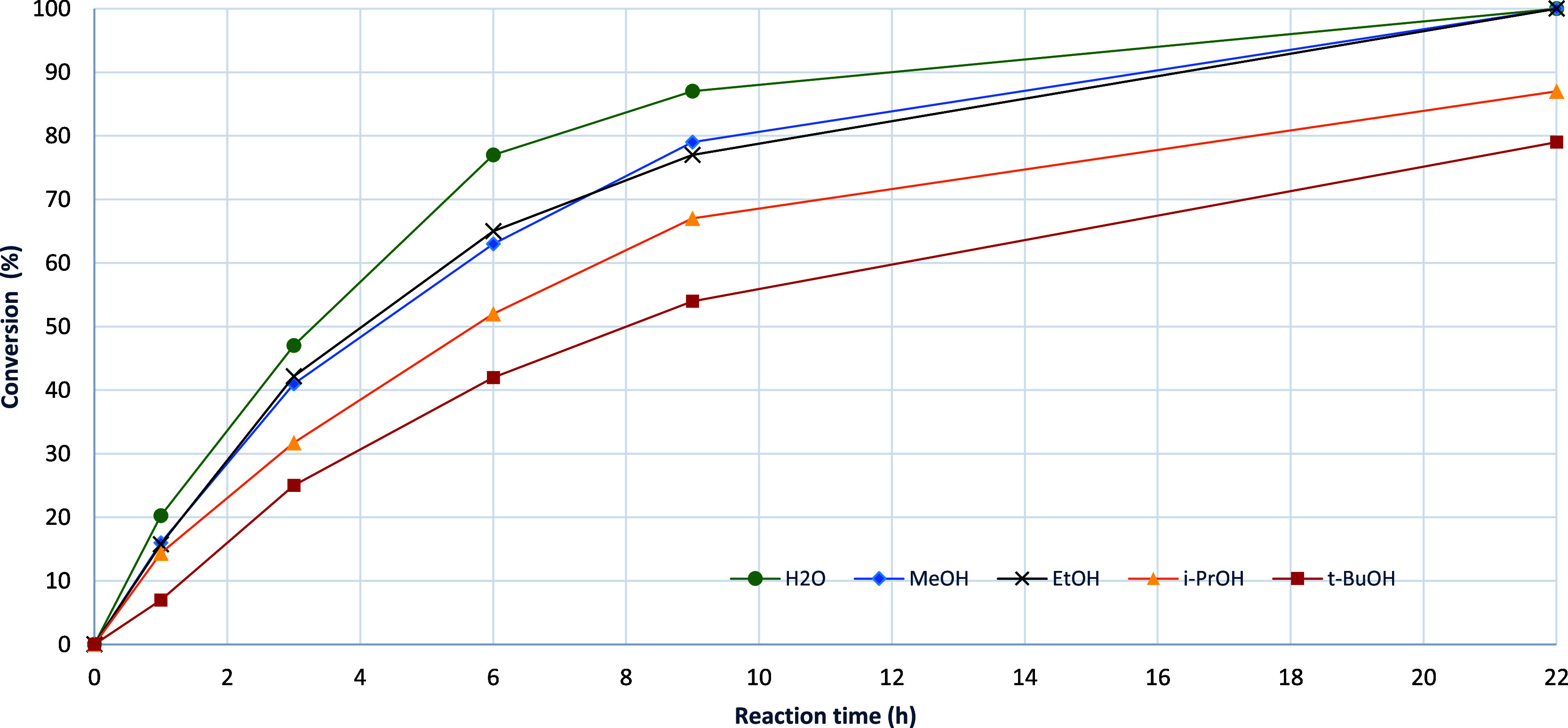
Effect of solvent type on formation of **3a** using 0.1 equiv of HCl. The reactions were performed at 60 °C.

2-Propanol (2-PrOH) has been the most popular solvent for these reactions, which might be reasonable from a solubility standpoint. We were curious as to how different solvents affected rate and product formation, since the choice of solvent can modulate the relative basicity of the reacting components,^28,29^ or stabilize the transition state and have an impact on the cost profile of the process. The same model reaction was therefore conducted in four different alcohols and water at 60 °C using 0.1 equiv of HCl. The reaction progress is shown in Figure 2.

From a practical viewpoint, the highest amount of product was obtained with water, MeOH or EtOH as solvent. Reactions in MeOH gave some solvolysis (5% after 22 h) which was not noted in the other solvents. The apparent higher rate for the more polar solvents could be explained by better ability to stabilize a polar transition state. However, the initial rate in 2-PrOH compared to that in MeOH/EtOH was rather similar. An alternative explanation is that that the most polar solvents have a better ability to hydrogen bond the released HCl, leading to a more favorable aniline-anilinium ion equilibrium position.

As reaction in water had the highest rate, and there is considerable risk related to combining alcoholic solvents with HCl,^30^ we proceeded with testing the effect of the HCl amount in the reaction. To increase the rate, the reaction temperature was raised from 60 to 80 °C. The results are shown in Table 1. The initial rate as measured after 20 min was dependent on the amount of acid. The reaction with 0.1 equiv of HCl (Table 2, entry 1) had a slower onset than the reaction with 1.0 equiv of HCl (entry 5), which after 20 min had a conversion >50%. Anyhow, all reactions reached full conversion after 6 h. Low levels of the solvolysis side-product **5** was detected in all cases. However, compound **5** has good water solubility, and the actual levels can be somewhat higher than that detected due to the extractive workup performed on the analytical NMR samples. For the reaction reported in entry 1, direct sampling without extraction confirmed that in this case the level of **5** was below detection level.

**Table 1:**
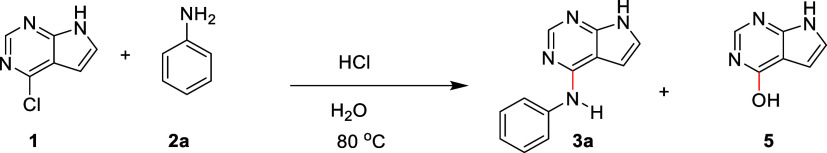
Effect of HCl Amount on Reaction Progress in Water and Formation **3a** and the Side-Product **5**

entry	HCl (equiv)	conv. 0.33 h (%)^a^	mole % after 6 h^b^		
			1	3a	5^c^
1	0.1	19	<1	>98	<1
2	0.2	28	<1	>98	<1
3	0.5	48	<1	98	1
4	0.8	59	<1	98	1
5	1.0	54	<1	98	1

a Conversion was measured by ^1^H NMR, conv (%) = 100 × [**3a** + **5**]/[**1** + **3a** + **5**].

b Mole % of **1**, **3a**, and **5**, < denoted an undetected compound.

c Actual levels are likely to be somewhat higher due to the extractive workup. A simulated extraction indicates that 60% of **5** is recovered by four extractions with EtOAc.

**Table 2:**
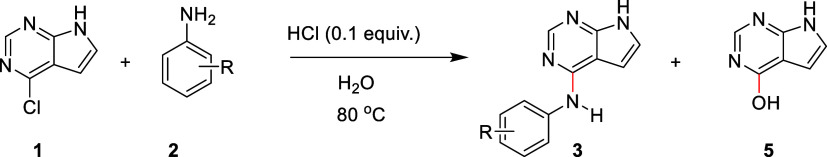
Substrate Scope in HCl-Promoted Amination of 4-Chloro-7*H*-pyrrolo[2,3-*d*]pyrimidine with Anilines^a^

entry	aniline	p*K*_a_^b^	conv. 1 h (%)^c^	reaction time (h)	mole (%)^d^			yield (%)	prod.
					1	3	5		
1	4-OEt (**2b**)	5.19	78	3	<1	>98	<1	94	**3b**
2	4-Bu (**2c**)	4.95	66	6	<1	>98	<1	88	**3c**
3	3,4-methylene-dioxy (**2d**)	4.46	74	3	<1	>98	<1	85	**3d**
4	4-F (**2e**)	4.65	77	6	<1	>98	<1	92	**3e**
5	H (**2a**)	4.58	68	6	<1	>98	<1	91	**3a**
6	3-OBn (**2f**)	ca. 4.2	86	6	<1	>98	<1	87	**3f**
7	3-ethyne (**2g**)	3.82	81	6	<1	>98	<1	83	**3g**
8	3-Cl (**2h**)	3.34	80	6	<1	>98	<1	81	**3h**
9	4-Br-3-F (**2i**)	2.73	84	3	<1	>98	<1	91	**3i**
10	4-NO_2_ (**2j**)	1.02	15	6	<1	97	3	88	**3j**
11	N-Me-4-F (**2k**)	ca. 4.9	15	22	4	96	<1	88	**3k**
12	2-OH (2**l**)	4.84	44	22	2	96	2	89	**3l**
13	2,6-(i-Pr)_2_ (**2m**)	4.51	0	22	83	0	17		**3m**
14	2-I (**2n**)	2.6	3	22	3	94	3	79	**3n**
15	2,4-Cl (**2o**)	2.0	11	22	<1	85	15	80	**3o**
16	2,4,5-Cl (**2p**)	1.09	0	22	73	15	12		**3p**
17	2,6-Cl (**2q**)	0.42	0	22	83	0	17		**3q**
18	2-NO_2_ (**2r**)	–0.31	0	22	78	5	17		**3r**
19	2-CF_3_, 4-NO_2_ (**2s**)	<0	0	22	85	0	15		**3s**
20	2,3,4,5,6-F (**2t**)	–0.28	0	22	82	3	15		**3t**

a Conversion data and mole (%) data is from 100 mg reactions, while isolated yields are from 500 mg reactions.

b The specific sources for the experimental and calculated p*K*_a_ values,^31−34^ are given in the Supporting Information, Table S3.

c Conversion of the amination after 1 h measure by ^1^H NMR: Conv. = 100 × [**3** + **5**]/[**1** + **3** + **5**], using signal from H-2.

d Mole % of **1**, **3**, and **5** at the termination point measured by ^1^H NMR. Values denoted as <1 means not detected. The levels of **5** can be somewhat underestimated by the analysis.

To minimize the use of chemicals, an amination process with no acid is preferable, but a slow nonreliable reaction onset can be problematic. A compromise is the use of 0.1 equiv of HCl. To show the applicability of this procedure a reaction was performed on a 500 mg scale giving 91% of the product **3a**.

### 2.3 Substrate Scope for Amination in Water

In terms of both reactivity and sustainability, the use of 0.1 equiv of HCl in water is very attractive. Another potential benefit with water as compared to alcohols as solvent is that solvent switch prior to extraction would not be needed. Therefore, we went on to evaluate the substrate scope of the amination by testing 19 more anilines having different p*K*_a_ and substitution patterns. The reactions were initially monitored on a 100 mg scale. This was followed by preparative reactions with 500 mg of 4-chloro-7*H*-pyrrolo[2,3-*d*]pyrimidine (**1**). Conversion data for the 100 mg reactions, isolated yields, and experimental/estimated p*K*_a_ of the anilines are shown in Table 2.

**Figure 3:**
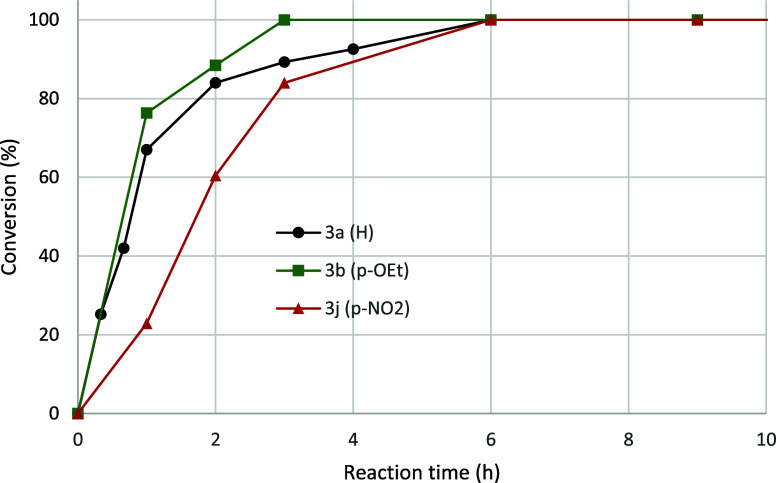
Comparison of reaction profiles for preparation of **3a** (black circles), **3b** (green squares), and **3j** (red triangles). Conv. (%) = 100 × (**3** + **5**)/ (**1** + **3** + **5**).

For the *para* and *meta*-substituted anilines (entries 1–10), all aminations reached full conversion within 6 h. Anilines with p*K*_a_ between 5.2–2.7 seemed to react somewhat faster than outside this range. However, 4-nitroaniline (p*K*_a_ = 1.02, entry 10) was also well suited as substrate, though more of the hydrolytic side-product **5** was formed. This is since water at lower pH becomes a competitive nucleophile. Certain functional groups can be labile under acidic conditions. Using 0.1 equiv of HCl, the pH went from 4.7 at start of the reaction to 2.0 at the end, which are mild conditions and debenzylation of **3f** and hydrolysis of the alkyne in **3g** were not observed. A comparison of the reaction profiles for **2a** (R = H), the most basic aniline **2b** (R = 4-OEt) and 4-nitroaniline (**2j**), is shown in Figure 3, where conversion is plotted vs reaction time.

**Figure 4:**
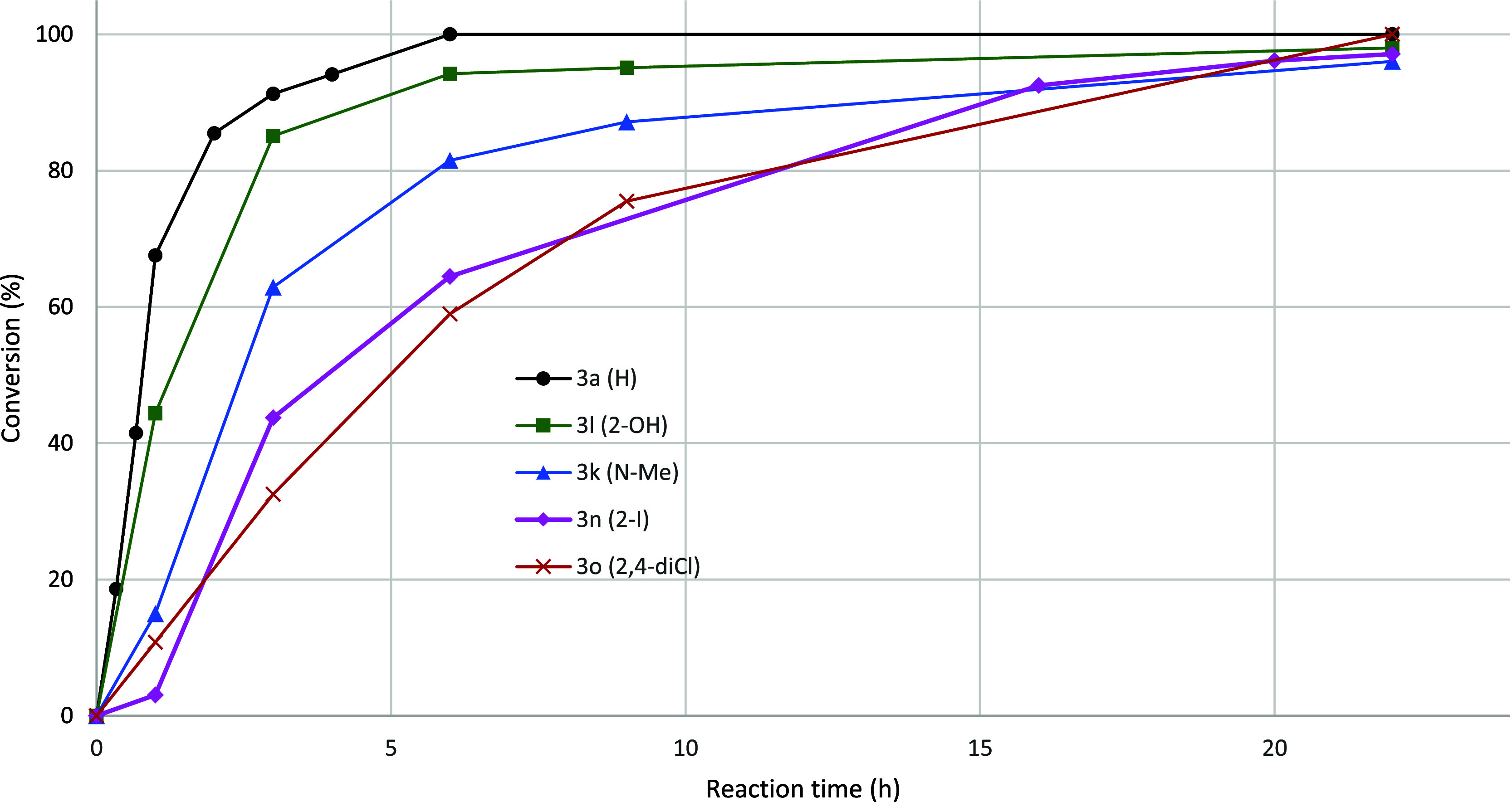
Comparison of reaction profiles for preparation of **3a** (black circles), **3l** (green squares), **3k**, (blue triangles), **3n** (pink tilted squares), and **3o** (red cross). Conv. (%) = 100 × (**3** + **5**)/ (**1** + **3** + **5**).

To evaluate steric effects, we also performed amination with 10 additional aniline derivatives (entries 11–20). *N*-Alkylation of anilines increase both their basicity and steric bulk. 4-Fluoro-*N*-methylaniline (**2k**, entry 11) reacted more slowly than aniline (**2a**), but proved to be a good substrate, indicating that some added bulk is allowed for. 2-Hydroxyaniline (**2l**, entry 12) showed very good reactivity. Somewhat slower reaction progress was seen for the bulky 2-iodoaniline (**2n**, entry 14) and the deiodinated side-product **3a** was also formed (8–10%). Similar deiodination have previously been observed.^35−37^ A few control experiments were performed to identify the reason for the deiodination. First, palladium contamination was ruled out. To test for radical type dehalogenation, degassing of solvent, and protection from day light was done, but this had no effect on the level of side-product **3a**. Further, when purified product **3n** was submitted to heating in water with 1.5 equiv of HCl, no deiodination took place in 22 h. In contrast, 2-iodoaniline (**2n**) was found to be unstable and provide aniline (**2a**). When we treated **2n** in the absence of **1** with more HCl (1.5 equiv) for 22 h also, 2,4-diiodoaniline and 2,6-diiodoaniline were formed, indicating that disproportionation is occurring under acidic conditions as seen by others.^38,39^ 2,4-Dichloroaniline (**2o**) with a lower p*K*_a_ had reactivity in line with that of 2-iodoaniline (**2n**). Figure 4 shows conversion vs time for reactions toward **3l**, **3k**, **3n**, and **3o** compared with that of the parent compound **3a**.

Introducing two *ortho*-substituents or combining *ortho*-substituents with a low p*K*_a_ (entries 13 and 16–20) prevents efficient amination and minimal production of product was seen. For these anilines with low nucleophilicity, water becomes a competing nucleophile, giving higher amounts of the side-product **5.**

Preparative reactions were performed with 500 mg of **1** and 1.1 equiv of the anilines **2a**–**l**, **2n**, and **2o**. At the onset of the reactions, the mixture is a slurry which dissolves as more acid is produced. Then, when sufficient amount of product is formed, it starts to precipitate. Thus, at larger scales, mechanical stirring should be used. The products were purified by silica-gel column chromatography, giving 80–94% isolated yield (average 87%). The moderate yield for the *ortho*-iodo analogue **3n** is due to side-product formation (comp. **3a**), while the reaction to form the 2,4-dichloro analogue **3o** produced more of the hydrolytic side-product **5**. To conclude, amination of 4-chloro-7*H*-pyrrolo[2,3-*d*]pyrimidine (**1**) proceeds well with *meta* and *para*-substituted anilines, with p*K*_a_ ranging from 5.3 to 1.0. Steric hindrance introduced at the nucleophilic nitrogen, and *ortho*-chloro and *ortho*-iodo substitution leads to lower rate, but acceptable reactions if the p*K*_a_ of the aniline is not too low. In addition to cost savings, the use of water instead of 2-PrOH, prevents the formation of 2-chloropropane.^40,41^ Some of these reactions might also proceed well with lower amount of acid, although with a somewhat slow onset. For instance, the parent compound **3a** (R = H) can be formed without acid added, while the 2-iodo analogue **3n** was not formed without acid. Finally, these compounds can also be isolated by precipitation directly from the reaction mixture.

On full scale production, the use of an even cheaper proton source than HCl could be of interest. Therefore, the amination of **1** with aniline was tested with 0.05 equiv of H_2_SO_4,_, and the reaction proceeded well (Figure S2, Supporting Information). Alternatively, in educational laboratories, less hazardous acids might be advantageous, and 0.1 equiv of acetic acid also efficiently promote this amination (Figure S2, Supporting Information). DMF is a commonly employed solvent in nucleophilic aromatic substitution. In DMF only, the conversion rate was lower than for the reaction in water, while the use of DMF/water (1:1 by vol %) re-established a good rate (Figure S3, Supporting Information). Thus, DMF is a possible cosolvent for substrates with low water solubility.

### 2.4 Amination of Other Heterocycles with Aniline

We then evaluated amination of seven other fused pyrimidines (compounds **6**-**12**, Table 3) with aniline (**2a**). 4-Chloroquinoline (**6**, entry 1) reacted much faster than the pyrrolopyrimidine **1**, and in amination at 80 °C without HCl, the reaction gave the product **13** alongside the hydrolytic side-product quinazolin-4-ol. Formation of the latter was suppressed by lowering the reaction temperature to 40 °C, for which the reaction went to completion in 1 h giving 85% isolated yield of **13**. The whole reaction occurred in a slurry. 4-Chlorothieno[2,3-*d*]pyrimidine (**7**, entry 2) also reacted fast without HCl at 80 °C, giving 89% yield of **14** after a reaction time of 1 h. ^1^H NMR of the crude product did not show the hydrolytic side-product. Previously, this compound has been prepared using InCl_3_ as promotor (70% yield),^22^ and reaction at 150 °C in DMF (66% yield).^42^ 6-Chloropurine (**8**, entry 3) when aminated without addition of HCl, had a slow, onset, but after 24 h, full conversion was seen, without any sign of the hydrolytic side-product. In contrast, the use of 0.1 equiv. HCl enabled this amination to be complete in 1 h (86% isolated yield). Thus, the reactivity of the heterocycles follows the trend quinazoline > thienopyrimidine > purine > pyrrolopyrimidine. 4-Chloro-5-iodo-7*H*-pyrrolo[2,3-*d*]pyrimidine (**9**) (entry 4) proved unsuited for the reaction, since **9** proceeded to give the deiodinated structure **3a** as the main product, alongside other components probably originating from disproportionation reactions as seen for 2-iodoaniline (**2n**). Reaction with the rather lipophilic trimethylsilylethoxymethyl (SEM) protected pyrrolopyrimidine **10** (entry 6) in water with 0.1 equiv. HCl did not give the intended product **16**, but small amounts of the deprotected derivative **3a**. However, in 2-PrOH the reaction after 18 h gave 94% isolated yield of **16**. Highly crystalline compound with limited water solubility generally represents a challenge for reactions in water. We tested two such pyrrolopyrimidines **11** (mp. 245–247 °C) and **12** (mp. > 300 °C) in acid-promoted amination to the corresponding products **17** and **18** (entries 6 and 7). In water, the pyrrolopyrimidines **11** and **7** were completely insoluble and the amination proceeded very slowly. After 3 days, 60% conversion was observed for the least crystalline **11**, while no product formation could be detected using the bromo containing **12**. For these substrates, a change to 2-PrOH resulted in full conversion after 22 h, giving compound **17** and **18** in 82 and 87% isolated yield, respectively.

**Table 3:**
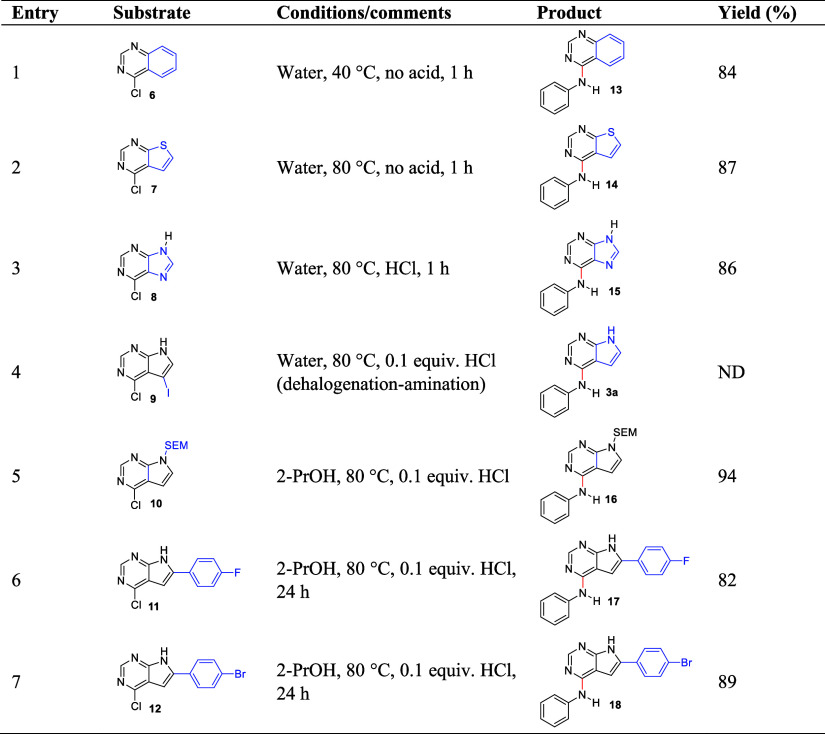
Amination of Different Fused Pyrimidines with Anilines in Water under Different Conditions

Finally, 1-fluoro-4-nitrobenzene, an excellent substrate in nucleophilic aromatic substitution, was tested as substrate in this amination. Only trace amounts of the expected product were seen after 24 h using either water or *i*-PrOH as solvent (data not shown). Thus, the weakly basic pyrimidine nitrogens seem essential to promote the acid-catalyzed amination.

To conclude, different heterocycles can be aminated with anilines under acidic conditions, but, require modification of the reaction conditions to give optimal yield. Quinazolines are so reactive that the reaction temperature must be lowered, and no acid is needed at start, while reaction with purines have a slow onset and assistance from acid is highly beneficial. Highly lipophilic and crystalline compounds are best aminated in 2-PrOH.

Highly basic amines should be unsuited for amination under acidic conditions as they would be protonated and thus non-nucleophilic. Thus, for comparison with the aniline aminations, we also performed experiments under acidic conditions with such amines (Supporting Information, Table S3). In short, the use of aliphatic and benzylic amines in reaction with fused pyrimidines in the presence of protic acids leads to low conversion to product. However, water can be an excellent solvent for these reactions in the absence of acid.

## 3 Conclusions

Nucleophilic aromatic substitution on heterocyclic chlorides with anilines is an important reaction in medicinal chemistry research, and for these substrates, the use of acid-promoted reactions in water appear attractive both in terms of simplicity, yield, and sustainability. For suitable substrate pairs, considerable cost savings can be achieved using water and HCl instead of organic solvents and more complicated promotors or catalysts. In these aminations, the amount of acid should be kept low to minimize the competing solvolysis. Thus, in reaction between 4-chloro-7*H*-pyrrolo[2,3-*d*]pyrimidine (**1**) and aniline, 0.1 equiv of HCl is sufficient, and the reaction proceeds with higher rate in water than in short chain alcohols. Sulfuric acid or acetic acid can also be used as catalyst in this these transformations. The substrate scope of the amination in water was evaluated with 20 aniline derivatives. The amination of **1** proceeds well with *meta* and *para*-substituted anilines, with p*K*_a_ ranging from 1.0 to 5.3. Steric hindrance introduced at the nucleophilic nitrogen, and *ortho*-chloro and iodo substitution leads to lower rate, but still acceptable reactions. Other heterocycles can also be aminated in water, though 4-chloroquinazoline and 4-chlorothieno[2,3-*d*]pyrimidine are more reactive and does not need to be kick started using acid. Limitations to the use of water/HCl amination includes *ortho*-substituted anilines with p*K*_a_ below 1, 2,6-disubstituted anilines, and lipophilic and crystalline pyrrolopyrimidines. Amination of the latter two substrate classes is best performed with 2-PrOH as solvent. Although less attractive from a sustainability perspective, DMF can be employed as a cosolvent in these transformations. Deiodination was observed for reaction involving 2-iodoaniline as nucleophile and a 5-iodinated pyrrolopyrimidine as electrophile, and special care and attention should be given to such substrates. Aliphatic and benzylic amines due to their high basicity cannot be reacted with aryl halides under acidic conditions. However, these aminations proceed with good rate in water without acid.

## 4 Experimental Section

### 4.1 Chemicals and Analysis

All solvents and most reagents used in the project were purchased from VWR and Merck. 4-Chloro-7*H*-pyrrolo[2,3-*d*]pyrimidine (**1**) was obtained from 1 Click Chem, while 4-chloroquinazoline (**6**) and 6-chloropurine (**8**) were from Merck. The substrates 4-chloro-5-iodo-7*H*-pyrrolo[2,3-*d*]pyrimidine (**9**),^43^ 4-chloro-7-((2-(trimethylsilyl)ethoxy)methyl)-7*H*-pyrrolo[2,3-*d*]pyrimidine (**10**),^5^**11**, and **12**,^44^ were prepared, as previously described. Reference samples for the hydrolytic side-products **5** and quinazolin-4-ol were from Merck, while thieno[2,3-*d*]pyrimidin-4(3*H*)-one was made in-house.^45^ Silica-gel chromatography was performed using silica-gel 60A purchased from VWR with a pore size 40–63 um. ^1^H- and ^13^C NMR spectra were recorded using a Bruker Advance III HD NMR spectrometer from Nanaobay electronics with a Smartprobe 5 mm probe head, operating at 400 and 600 MHz for proton, and carbon spectra at 100 and 150 MHz, respectively. All ^19^F NMR chemical shifts are relative to internal hexafluorobenzene in DMSO*-d*_*6*_ at δ = −163.0 ppm. Samples were mainly analyzed in DMSO-*d*_*6*_. ^1^H and ^13^C NNR chemical shifts are in ppm relative to the DMSO-*d*_*6*_ solvent peak at 2.50 and 39.5 ppm, respectively. High resolution mass spectroscopy (HRMS) was performed using a WaterTM’s Synapt G2-S Q-TOF instrument. Samples were ionized by electrospray ionization (ESI/70 eV) and analyzed using an atmospheric solids analysis probe (ASAP). Calculated exact mass and spectra processing was done by WatersTM Software (Masslynx V4.1 SCN871).

### 4.2 General Synthetic Methods

#### 4.2.1 General Procedure A: Test Amination (100 mg scale)

4-Chloro-7*H*-pyrrolo[2,3-*d*]pyrimidine (**1**, 100 mg, 0.651 mmol, 1.0 equiv) was mixed with the appropriate aniline (1.0 equiv) and in the specified solvent (5 mL), and HCl (0–5 equiv) were added. The reaction mixtures were stirred for up to 22 h at 60 or 80 °C, with 4–5 samples taken out for ^1^H NMR analysis. The samples withdrawn were diluted with EtOAc (1–2 mL) and aq. NaHCO_3_ (1–2 mL) was added. After phase separation, drying, and concentration, the residue was dissolved in DMSO-*d*_*6*_ and analyzed by ^1^H NMR spectroscopy. Integration of the pyrrolopyrimidine H-2 protons were used to estimate levels of substrate, product, and side-product. After cooling to room temperature, the reaction mixtures were suspended in sat. Na_2_CO_3_ (aq., 2 mL) and vacuum filtered, washed with water, and dried. The compounds were purified by silica-gel column chromatography to confirm the identity of the product.

#### 4.2.2 General Procedure B: 500 mg Scale Amination in Water

4-Chloro-7*H*-pyrrolo[2,3-*d*]pyrimidine (500 mg, 3.26 mmol, 1 equiv) and the appropriate anilines (1.1 equiv) were mixed with H_2_O (25 mL) and HCl (0.61 M, 0.1 equiv). The reaction mixtures were stirred at 80 °C for 3–22 h. After cooling to room temperature, the reaction mixtures were suspended in sat. Na_2_CO_3_ (aq. Ten mL) and the formed solid was isolated by filtration. To recover more material, the filtrates were extracted with EtOAc (4 × 30 mL). The combined organic phases were dried with brine (2 × 20 mL) and anhydrous Na_2_SO_4_, followed by filtration and concentration in vacuo. The filtrate and precipitate were combined and dried in vacuo. The crude product was immobilized on Celite and purified using silica-gel flash chromatography as specified for each compound.

### 4.3 Isolated Materials

#### 4.3.1 *N*-Phenyl-7*H*-pyrrolo[2,3-*d*]pyrimidin-4-amine (**3a**)

The compound was prepared as described in procedure B, starting with aniline. The reaction time was 6 h The crude material was purified using gradient silica-gel flash chromatography (*n*-pentane/EtOAc, 1:2, *R*_f_ = 0.21, → EtOAc). This yielded 626 mg (2.98 mmol, 91%) as a white powder, mp. 240–243 °C (lit,^12^ 241 °C). ^1^H NMR (400 MHz, DMSO-*d*_*6*_) δ 11.74 (s, 1H), 9.28 (s, 1H), 8.27 (s, 1H), 7.93–7.85 (m, 2H), 7.38–7.28 (m, 2H), 7.23 (dd, *J* = 3.5, 2.3 Hz, 1H), 7.01 (tt, *J* = 7.3, 1.2 Hz, 1H), 6.78 (dd, *J* = 3.5, 1.8 Hz, 1H). The ^1^H NMR correspond to that found previously.^15^

#### 4.3.2 *N*-(4-Ethoxyphenyl)-7*H*-pyrrolo[2,3-*d*]pyrimidin-4-amine (**3b**)

The compound was synthesized on a 500 mg scale as described in procedure B using 4-ethoxyaniline. The reaction time was 9 h. The crude product was immobilized on Celite and purified using silica-gel flash chromatography (EtOAc/*n*-pentane, 2:1, *R*_f_ = 0.16). This gave 781 mg (3.07 mmol, 94%) of a white powder, mp. 240–242 °C (lit,^12^ 241–242 °C), ^1^H NMR (400 MHz, DMSO-*d*_*6*_) δ 11.66 (s, 1H), 9.13 (s, 1H), 8.20 (s, 1H), 7.70 (d, *J* = 9.0 Hz, 2H), 7.18 (d, *J* = 3.5 Hz, 1H), 6.91 (d, *J* = 9.0 Hz, 2H), 6.67 (d, *J* = 3.4 Hz, 1H), 4.00 (q, *J* = 6.9 Hz, 2H), 1.32 (t, *J* = 6.9 Hz, 3H). ^1^H NMR correspond to those previously described.^15,22^

#### 4.3.3 *N*-(4-Butylphenyl)-7*H*-pyrrolo[2,3-*d*]pyrimidin-4-amine (**3c**)

The compound was prepared as described in procedure B, starting with 4-butylaniline. The reaction time was 9 h. The crude material was purified using silica-gel flash chromatography (EtOAc/*n*-pentane, 2:1, *R*_f_ = 0.35). This gave 764 mg (2.87 mmol, 88%) of a white powder, mp. 195–197 °C. ^1^H NMR (400 MHz, DMSO-*d*_*6*_) δ 11.71 (s, 1H), 9.21 (s, 1H), 8.25 (s, 1H), 7.80–7.72 (m, 2H), 7.21 (dd, *J* = 3.5, 2.2 Hz, 1H), 7.18–7.11 (m, 2H), 6.76 (dd, *J* = 3.5, 1.8 Hz, 1H), 2.55 (t, *J* = 7.7 Hz, 2H), 1.62–1.50 (m, 2H), 1.39–1.25 (m, 2H), 0.91 (t, *J* = 7.3 Hz, 3H); ^13^C NMR (101 MHz, DMSO-*d*_*6*_) δ 154.1, 151.3, 151.3, 138.4, 136.5, 128.7 (2C), 122.4, 121.0 (2C), 104.0, 99.3, 34.7, 33.8, 22.2, 14.3; IR (neat, cm^–1^): 3172, 2951–2924, 2850, 1612, 1574, 1537, 1512, 1433, 1346, 1312, 898, 791; HRMS (ES+, *m*/*z*): found 267.1614, calcd. for C_16_H_19_N_4_, [M + H]^+^, 267.1610.

#### 4.3.4 *N*-(Benzo[*d*][1,3]dioxol-5-yl)-7*H*-pyrrolo[2,3-*d*]pyrimidin-4-amine (**3d**)

The compound was synthesized on a 500 mg scale as described in procedure B, using benzo[*d*][1,3]dioxol-5-amine. The reaction time was 22 h. The crude product was purified using flash silica-gel chromatography (EtOAc/*n*-pentane, 4:1, *R*_f_ = 0.20, → 100% EtOH, 100% → EtOAc/MeOH, 10:1). This yielded 701 mg (2.77 mmol, 85%) of a light red powder, mp. 282 °C (decomp.) (lit,^17^ 282–283 °C), ^1^H NMR (400 MHz, DMSO-*d*_*6*_) δ 11.71 (s, 1H), 9.18 (s, 1H), 8.23 (s, 1H), 7.59 (d, *J* = 2.2 Hz, 1H), 7.23–7.19 (m, 2H), 6.89 (d, *J* = 8.4 Hz, 1H), 6.71 (dd, *J* = 3.5, 1.9 Hz, 1H), 6.00 (s, 2H). The spectroscopic data correspond to those previously reported.^22^

#### 4.3.5 *N*-(4-Fluorophenyl)-7*H*-pyrrolo[2,3-*d*]pyrimidin-4-amine (**3e**)

The compound was prepared as described in procedure B, starting with 4-fluoroaniline. The reaction time was 6 h. The crude material was purified using silica-gel flash chromatography (EtOAc/*n*-pentane, 4:1, *R*_f_ = 0.23, → EtOAc). This gave 683 mg (3.00 mmol, 92%) of a white powder, mp. 251–253 °C (lit,^39^ 253 °C). ^1^H NMR (400 MHz, DMSO-*d*_*6*_) δ 11.76 (s, 1H), 9.34 (s, 1H), 8.27 (s, 1H), 7.92–7.86 (m, 2H), 7.23 (d, *J* = 3.5 Hz, 1H), 7.20–7.13 (m, 2H), 6.76 (d, *J* = 3.4 Hz, 1H). The ^1^H NMR correspond that found in the literature.^46^

#### 4.3.6 *N*-(3-(Benzyloxy)phenyl)-7*H*-pyrrolo[2,3-*d*]pyrimidin-4-amine (**3f**)

The compound was synthesized on a 500 mg scale as described in procedure B, using 3-benzyloxyaniline. The reaction time was 6 h. The crude product was immobilized on Celite and purified using flash chromatography (EtOAc/*n*-pentane, 4:1, *R*_f_ = 0.25, → EtOAc). This yielded 896 mg (2.83 mmol, 87%) of a light brown powder, mp. 194–196 °C, ^1^H NMR (400 MHz, DMSO-*d*_*6*_) δ 11.77 (s, 1H), 9.27 (s, 1H), 8.30 (s, 1H), 7.79 (t, *J* = 2.3 Hz, 1H), 7.52–7.29 (m, 6H), 7.27–7.18 (m, 2H), 6.81 (dd, *J* = 3.5, 1.9 Hz, 1H), 6.67 (dd, *J* = 8.2, 2.6, 1H), 5.11 (s, 2H); ^13^C NMR (101 MHz, DMSO-*d*_*6*_) δ 159.1, 153.9, 151.3, 151.2, 142.2, 137.7, 129.6, 128.9 (2C), 128.3, 128.2 (2C), 122.7, 113.1, 108.5, 107.4, 104.3, 99.2, 69.6; IR (neat, cm^–1^): 3179, 3061, 2924–2823, 1609, 1579, 1551, 1508, 1433, 1306, 1239, 1046, 1029, 824; HRMS (ES+, *m*/*z*): found 317.1407, calcd. for C_19_H_17_N_4_O, [M + H]^+^, 317.1402.

#### 4.3.7 *N*-(3-Ethynylphenyl)-7*H*-pyrrolo[2,3-*d*]pyrimidin-4-amine (**3g**)

The compound was synthesized on a 500 mg scale as described in general procedure B, using 3-ethynylaniline. The reaction time was 6 h. The crude product was purified using flash chromatography (EtOAc/*n*-pentane, 4:1, *R*_f_ = 0.23). This yielded 627 mg (2.77 mmol, 83%) of a white powder, mp. 228–230 °C, ^1^H NMR (400 MHz, DMSO-*d*_*6*_) δ 11.81 (s, 1H), 9.40 (s, 1H), 8.32 (s, 1H), 8.16 (t, *J* = 2.0 Hz, 1H), 7.91 (ddd, *J* = 8.4, 2.4, 1.0 Hz, 1H), 7.34 (t, *J* = 7.9 Hz, 1H), 7.26 (d, *J* = 3.5 Hz, 1H), 7.11 (dt, *J* = 7.6, 1.3 Hz, 1H), 6.80 (d, *J* = 3.5 Hz, 1H), 4.15 (s, 1H); ^13^C NMR (101 MHz, DMSO-*d*_*6*_) δ 153.7, 151.4, 151.1, 141.2, 129.4, 125.5, 123.2, 122.9, 122.2, 121.0, 104.3, 99.2, 84.3, 80.7; HRMS (ES+, *m*/*z*): found 235.0987, calcd. for C_14_H_11_N_4_, [M + H]^+^, 235.0984. Reference spectra has not been found.

#### 4.3.8 *N-*(3-Chlorophenyl)-7H-pyrrolo[2,3-*d*]pyrimidin-4-amine (**3h**)

The compound was prepared as described in procedure B, starting with 3-chloroaniline. The reaction time was 6 h. The crude material was purified using silica-gel flash chromatography (EtOAc/*n*-pentane, 4:1, *R*_f_ = 0.27). This gave 696 mg (2.69 mmol, 81%) of a white powder, mp. 226–227 °C (lit,^39^ 227 °C); ^1^H NMR (400 MHz, DMSO-*d*_*6*_) δ 11.85 (s, 1H), 9.46 (s, 1H), 8.35 (s, 1H), 8.22 (t, *J* = 2.1 Hz, 1H), 7.81 (dd, *J* = 8.2, 2.4 Hz, 1H), 7.35 (t, *J* = 8.1 Hz, 1H), 7.28 (d, *J* = 3.4 Hz, 1H), 7.03 (dd, *J* = 7.8, 2.4 Hz, 1H), 6.82 (d, *J* = 3.4 Hz, 1H). The ^1^H NMR correspond that found in the literature.^46^

#### 4.3.9 *N*-(4-Bromo-3-fluorophenyl)-7*H*-pyrrolo[2,3-*d*]pyrimidin-4-amine (**3i**)

The compound was synthesized on a 500 mg scale as described in procedure B. The reaction time was 3 h. The crude product was purified using flash silica-gel chromatography (EtOAc/*n*-pentane, 1:1, *R*_f_ = 0.28 → EtOAc). This yielded 908 mg (2.96 mmol, 91%) of a white solid, mp. 308–309 °C, ^1^H NMR (400 MHz, DMSO-*d*_*6*_) δ 11.88 (s, 1H), 9.61 (s, 1H), 8.37 (s, 1H), 8.26 (dd, *J* = 12.2, 2.4 Hz, 1H), 7.67–7.56 (m, 2H), 7.30 (dd, *J* = 3.6, 2.0 Hz, 1H), 6.82 (dd, *J* = 3.5, 1.5 Hz, 1H); ^13^C NMR (101 MHz, DMSO-*d*_*6*_) δ 156.8 (d, *J* = 241.0 Hz), 152.9, 151.0, 150.5, 141.9 (d, *J*= 10.6 Hz), 132.8 (d, *J* = 1.3 Hz), 122.9, 116.9 (d, *J* = 21.3 Hz), 107.5 (d, *J* = 27.5 Hz) 104.1, 98.6 (d, *J* = 21.3 Hz), 98.57; ^19^F NMR (376 MHz, DMSO-*d*_*6*_) δ −107.97 (dd, *J* = 12.2, 6.8 Hz); HRMS (ES+, *m*/*z*): found 307.0000, calcd. for C_12_H_9_N_4_FBr [M + H]^+^, 306.9995.

#### 4.3.10 *N*-(4-Nitrophenyl)-7*H*-pyrrolo[2,3-*d*]pyrimidin-4-amine (**3j**)

The compound was prepared as described in procedure B, starting with 4-nitroaniline. The reaction time was 9 h. The crude material was purified using silica-gel flash chromatography (EtOAc/*n*-pentane, 2:1, *R*_f_ = 0.27 → EtOAc/MeOH, 10:1). This yielded 732 mg (2.87 mmol, 88%) of a yellow powder, mp. 335–337 °C (lit,^39^ 331 °C). ^1^H NMR (400 MHz, DMSO-*d*_*6*_) δ 11.99 (s, 1H), 9.99 (s, 1H), 8.44 (s, 1H), 8.25 (s, 4H), 7.37 (dd, *J* = 3.5, 2.3 Hz, 1H), 6.89 (dd, *J* = 3.5, 1.9 Hz, 1H). The ^1^H NMR correspond that found in the literature.^46^

#### 4.3.11 *N*-(4-Fluorophenyl)-*N*-methyl-7*H*-pyrrolo[2,3-*d*]pyrimidin-4-amine (**3k**)

The compound was prepared as described in general procedure B, starting with *N*-methyl-4-fluoroaniline. The reaction time was 22 h. The crude material was purified using silica-gel flash chromatography (gradient, EtOAc/*n*-pentane, 4:1, *R*_f_ = 0.10, → EtOAc). This gave 694 mg (2.87 mmol, 88%) of a white powder, mp. 250–252 °C; ^1^H NMR (400 MHz, DMSO-*d*_*6*_) δ 11.61 (s, 1H), 8.27 (s, 1H), 7.48–7.28 (m, 4H), 6.90 (d, *J* = 3.5 Hz, 1H), 4.66 (d, *J* = 3.5 Hz, 1H), 3.50 (s, 3H); ^13^C NMR (101 MHz, DMSO-*d*_*6*_) δ 160.3 (d, *J* = 244.1 Hz), 156.4, 151.8, 151.2, 142.5 (d, *J* = 1.0 Hz), 130.6 (d, *J* = 8.4 Hz, 2C), 121.5, 116.4 (d, *J* = 22.7 Hz, 2C), 103.3, 100.8, 39.4; ^19^F NMR (565 MHz, DMSO-*d*_*6*_) δ −114.95 – −115.04 (m); IR (neat, cm^–1^): 3189, 3114–3079, 2838, 1582, 1560, 1504, 1474, 1397, 1366, 1343, 1305, 1187, 895, 830; HRMS (ES+, *m*/*z*): found 243.1051, calcd. for C_13_H_12_N_4_F, [M + H]^+^, 243.1046.

#### 4.3.12 2-((7*H*-Pyrrolo[2,3-*d*]pyrimidin-4-yl)amino)phenol (**3l**)

The compound was isolated on a 500 mg scale as described in procedure B, using 2-aminophenol. The reaction time was 22 h. The crude product was immobilized on Celite and purified using flash silica-gel chromatography (gradient, EtOAc/*n*-pentane, 4:1, *R*_f_ = 0.21 → EtOAc). This yielded 650 mg (2.90 mmol, 89%) of a light-yellow powder, mp. 232–234 °C (lit,^17^ 233–235 °C); ^1^H NMR (400 MHz, DMSO-*d*_*6*_) δ 11.81 (s, 1H), 10.60 (s, 1H), 8.89 (s, 1H), 8.21 (s, 1H), 7.56 (dd, *J* = 7.9, 1.7 Hz, 1H), 7.21 (dd, *J* = 3.5, 2.2 Hz, 1H), 7.02 (td, *J* = 7.3, 1.7 Hz, 1H), 6.92 (dd, *J* = 8.1, 1.6 Hz, 1H), 6.84 (td, *J* = 7.5, 1.6 Hz, 1H), 6.70 (dd, *J* = 3.5, 1.6 Hz, 1H). The ^1^H NMR correspond to that previously reported.^22^

#### 4.3.13 *N*-(2-Iodophenyl)-7*H*-pyrrolo[2,3-*d*]pyrimidin-4-amine (**3n**)

The compound was prepared as described in procedure B, starting with 2-iodoaniline. The reaction time was 22 h. The crude product was immobilized on Celite and purified 3 times using flash silica-gel chromatography (CH_2_Cl_2_/acetone, 4:1, *R*_f_ = 0.20, → CH_2_Cl_2_/acetone, 1:1). This gave 865 mg (2.57 mmol, 79%) of an off-white solid, mp. 216–218 °C; ^1^H NMR (600 MHz, DMSO-*d*_*6*_) δ 11.69 (s, 1H), 9.03 (s, 1H), 8.12 (s, 1H), 7.94 (dd, *J* = 8.0, 1.5 Hz, 1H), 7.54 (dd, *J* = 7.9, 1.6 Hz, 1H), 7.43 (td, *J* = 7.6, 1.5 Hz, 1H), 7.16 (dd, *J* = 3.5, 2.1 Hz, 1H), 7.03 (td, *J* = 7.6, 1.6 Hz, 1H), 6.35 (dd, *J* = 3.4, 1.6 Hz, 1H); ^13^C NMR (150 MHz, DMSO-*d*_*6*_) δ 154.6, 151.1, 151.0, 141.2, 138.9, 128.8, 128.7, 127.6, 121.9, 102.9, 99.5.0, 98.8; IR (neat, cm^–1^): 3374, 1609, 1592, 1577, 1560, 1434, 1350, 1131, 1004, 897, 743; HRMS (ES+, *m*/*z*): found 336.9956, calcd. for C_12_H_10_N_4_I, [M + H]^+^, 336.9950.

#### 4.3.14 *N*-(2,4-Dichlorophenyl)-7*H*-pyrrolo[2,3-*d*]pyrimidin-4-amine (**3o**)

The compound was prepared as described in procedure B, starting with 2,4-dichloroaniline. The reaction time was 16 h. The crude material was purified using silica-gel flash chromatography (EtOAc/*n*-pentane, 1:1, *R*_f_ = 0.33 → EtOAc/MeOH, 10:1). This gave 728 mg (2.61 mmol, 80%) of a white powder. ^1^H NMR (400 MHz, DMSO-*d*_*6*_) δ 11.76 (s, 1H), 9.10 (s, 1H), 8.15 (s, 1H), 7.75 (d, *J* = 8.7 Hz, 1H), 7.70 (d, *J* = 2.4 Hz, 1H), 7.45 (dd, *J* = 8.6, 2.4 Hz, 1H), 7.22 (dd, *J* = 3.5, 2.3 Hz, 1H), 6.60 (dd, *J* = 3.5, 2.0 Hz, 1H); ^13^C NMR (101 MHz, DMSO-*d*_*6*_) δ 153.6, 151.5, 151.1, 142.6, 133.3, 130.5, 123.1, 121.8, 119.6, 118.6, 104.4, 99.1; IR (neat, cm^–1^): 3414, 3108–3067, 2852, 1620, 1593, 1584, 1480, 1457, 1418, 1355, 1283, 1135, 1092, 1051, 898, 822; HRMS (ES+, *m*/*z*): found 279.0208, calcd. for C_12_H_9_N_4_Cl_2_, [M + H]^+^, 279.0204.

#### 4.3.15 4-Ethoxy-7*H*-pyrrolo[2,3-*d*]pyrimidine (**4**)

The material was isolated following an amination of **1** with aniline in EtOH according to method A. Silica-gel flash chromatography (EtOAc/*n*-pentane, 2:1, *R*_f_ = 0.21) gave 5 mg (3.06 mmol) of a solid. ^1^H NMR (400 MHz, DMSO-*d*_*6*_) δ 11.99 (s, 1H), 8.34 (s, 1H), 7.33 (dd, *J* = 3.5, 2.3 Hz, 1H), 6.45 (dd, *J* = 3.4, 1.8 Hz, 1H), 4.52 (q, *J* = 7.1 Hz, 2H), 1.38 (t, *J* = 7.1 Hz, 3H); ^13^C NMR (101 MHz, DMSO-*d*_*6*_) δ 161.8, 152.5, 150.3, 124.0, 104.4, 97.8, 61.5, 14.5; HRMS (ASAP+, *m*/*z*): found 164.0827, calcd for for C_8_H_10_N_3_, (M+H)^+^, 164.0824.

#### 4.3.16 4-Chlorothieno[2,3-*d*]pyrimidine (**7**)

Thieno[2,3-*d*]pyrimidin-4(3*H*)-one (4.89 g, 32.1 mmol) and POCl_3_ (13 mL,) were mixed and agitated at 110 °C for 3.5 h, before being cooled to 22 °C. The mixture was poured into ice–water (300 mL) and neutralized with 5 M NaOH solution (95 mL). The solid formed was isolated by filtration and thoroughly washed with water. Drying of the solid under reduced pressure gave off a beige solid which was purified by silica-gel column chromatography (CH_2_Cl_2_), giving 4.77 g (28.0 mmol, 87%) of a white solid, mp. 105–106 °C (lit,^47^ 105 °C); ^1^H NMR (400 MHz, DMSO-*d*_6_) δ 8.96 (s, 1H), 8.15 (d, *J* = 6.0, 1H), 7.60 (d, *J* = 6.0, 1H); ^1^H NMR corresponded with that reported.^48^

#### 4.3.17 *N*-Phenylquinazolin-4-amine (**13**)

4-Chloroquinazoline (**6**, 500 mg, 3.04 mmol 1. equiv) and aniline (1.1 equiv) were mixed with H_2_O (25 mL). The reaction mixture was stirred at 40 °C for 1 h. After cooling to room temperature, the reaction mixtures were suspended in sat. Na_2_CO_3_ (aq. 10 mL) and the formed solid was isolated by filtration. To recover more material, the filtrates were extracted with EtOAc (4 × 30 mL). The combined organic phases were dried with brine (2 × 20 mL) and anhydrous Na_2_SO_4_, followed by filtration and concentration in vacuo. The filtrate and precipitate were combined and dried in vacuo. The crude product was immobilized on Celite and purified using silica-gel flash chromatography (EtOAc/*n*-pentane, 2:3, R_f_ 0.10). This gave 569 mg (2.57 mmol, 85%) of a white solid, mp. 221–223 °C (lit,^49^ 226–227 °C). ^1^H NMR (400 MHz, DMSO-*d*_*6*_) δ 9.80 (s, 1H), 8.63–8.54 (m, 2H), 7.91–7.76 (m, 4H), 7.63 (t, *J* = 7.6 Hz, 1H), 7.40 (t, *J* = 7.8 Hz, 2H), 7.13 (t, *J* = 7.4 Hz, 1H). The ^1^H NMR matched well that reported at 500 MHz.^49^

#### 4.3.18 *N-*Phenylthieno[2,3-*d*]pyrimidin-4-amine (**14**)

4-Chlorothieno[2,3-*d*]pyrimidine (**7**, 500 mg, 2.93 mmol 1. equiv) and aniline (1.1 equiv) were mixed with H_2_O (25 mL). The reaction mixture was stirred at 80 °C for 1 h. After cooling to room temperature, the reaction mixtures were suspended in sat. Na_2_CO_3_ (aq. 10 mL) and the formed solid was isolated by filtration. To recover more material, the filtrates were extracted with EtOAc (4 × 30 mL). The combined organic phases were dried with brine (2 × 20 mL) and anhydrous Na_2_SO_4_, followed by filtration and concentration in vacuo. The filtrate and precipitate were combined and dried in vacuo. The crude product was immobilized on Celite and purified using silica-gel flash chromatography (EtOAc/*n*-pentane, 1:4→ 1:1, *R*_f_ = 0.43). This gave 590 mg (2.60 mmol, 89%) of a white solid, mp. 173–175 °C (lit,^22^ 175–176 °C). ^1^H NMR (400 MHz, DMSO-*d*_*6*_) δ 9.65 (s, 1H), 8.50 (s, 1H), 7.90 (d, *J* = 6.0 Hz, 1H), 7.88–7.80 (m, 2H), 7.72 (d, *J* = 6.0 Hz, 1H), 7.43–7.33 (m, 2H), 7.14–7.06 (m, 1H). ^1^H NMR corresponded with that reported previously.^22^

#### 4.3.19 *N*-Phenyl-9*H*-purin-6-amine (**15**)

The compound was prepared as described in general procedure B, starting with 6-chloropurine (500 mg, 3.24 mmol) and aniline (1.1 equiv). The reaction time was 1 h. The crude material was purified using silica-gel flash chromatography (CH_2_Cl_2_/MeOH, 95:5, *R*_f_ = 0.14). This gave 585 mg (2.77 mmol, 85%) of a white solid, mp. 285–287 °C (lit,^50^ 278 °C). ^1^H NMR (400 MHz, DMSO-*d*_*6*_) δ 13.08 (s, 1H), 9.73 (s, 1H), 8.38 (s, 1H), 8.28 (s, 1H), 7.99–7.92 (m, 2H), 7.36–7.28 (m, 2H), 7.02 (t, *J* = 7.3, 1H). ^1^H NMR matched well with that reported previously at 200 MHz.^50^

#### 4.3.20 *N*-Phenyl-7-((2-(trimethylsilyl)ethoxy)methyl)-7*H*-pyrrolo[2,3-*d*]pyrimidin-4-amine (**16**)

4-Chloro-7-((2-(trimethylsilyl)ethoxy)methyl)-7*H*-pyrrolo[2,3-*d*]pyrimidine (**10**, 500 mg, 1.76 mmol, 1.0 equiv) was mixed with aniline (1.1 equiv), and 2-PrOH (25 mL) and HCl (0.61 M, 0.1 equiv) were added. The reaction mixture was stirred for 18 h at 80 °C. After cooling to room temperature, the reaction mixture was suspended in sat. Na_2_CO_3_ (aq., 10 mL) and vacuum filtered. To recover more material, the filtrate was extracted with EtOAc (4 × 50 mL). The combined organic phases were dried with brine (2 × 5 mL) and anhydrous Na_2_SO_4_, followed by filtration and concentration in vacuo. Both filtrate and precipitate were combined and dried in vacuo. The crude product was immobilized on Celite and purified using silica-gel flash chromatography (*n*-pentane/EtOAc, 4:1, *R*_f_ = 0.11, → *n*-pentane/EtOAc, 1:1). This gave 564 mg (1.66 mmol, 94%) of a white solid, mp. 137–138 °C. ^1^H NMR (600 MHz, DMSO-*d*_*6*_) δ 9.41 (s, 1H), 8.35 (s, 1H), 7.89 (d, *J* = 8.0 Hz, 2H), 7.40 (d, *J* = 3.6 Hz, 1H), 7.34 (t, *J* = 7.7 Hz, 2H), 7.03 (t, *J* = 7.3 Hz, 1H), 6.88 (d, *J* = 3.5 Hz, 1H), 5.55 (s, 2H), 3.51 (t, *J* = 8.0 Hz, 2H), 0.82 (t, *J* = 7.9 Hz, 2H), −0.09 (d, *J* = 1.4 Hz, 9H); ^13^C NMR (151 MHz, DMSO-*d*_*6*_) δ 153.7, 151.2, 150.5, 140.1, 128.5 (2C), 125.5, 122.2, 120.4 (2C), 103.8, 99.5, 72.2, 65.4, 17.1, −1.4 (3C); IR (neat, cm^–1^): 3262, 3184, 3106, 1612, 1579, 1558, 1467, 1446, 1303, 1233, 1092–1068, 833, 752, 738, 722.

#### 4.3.21 6-(4-Fluorophenyl)-*N*-phenyl-7*H*-pyrrolo[2,3-*d*]pyrimidin-4-amine (**17**)

4-Chloro-6-(4-fluorophenyl)-7*H*-pyrrolo[2,3-*d*]pyrimidine (**7**, 100 mg, 0.404 mmol, 1.0 equiv) was mixed with aniline (1.1 equiv), and 2-PrOH (5 mL) and HCl (0.61 M, 0.1 equiv) were added. The reaction mixture was stirred for 22 h at 80 °C. After cooling to room temperature, the reaction mixture was suspended in sat. Na_2_CO_3_ (aq., 2 mL) and vacuum filtered. To recover more material, the filtrate was extracted with EtOAc (4 × 10 mL). The combined organic phases were dried with brine (2 × 5 mL) and anhydrous Na_2_SO_4_ followed by filtration and concentration in vacuo. Both filtrate and precipitate were combined and dried in vacuo. The crude product was immobilized on Celite and purified using silica-gel flash chromatography (CH_2_Cl_2_/acetone, 4:1, *R*_f_ = 0.17, → CH_2_Cl_2_/acetone, 1:1). This gave 101 mg (0.331 mmol, 82%) of a white solid, mp. 323–326 °C; ^1^H NMR (600 MHz, DMSO-*d*_*6*_) δ 12.30 (s, 1H), 9.38 (s, 1H), 8.30 (s, 1H), 7.94–7.89 (m, 2H), 7.89–7.83 (m, 2H), 7.38–7.29 (m, 4H), 7.16 (s, 1H), 7.02 (tt, *J* = 7.3, 1.2 Hz, 1H); ^13^C NMR (150 MHz, DMSO-*d*_*6*_) δ 161.6 (d, *J* = 244.9 Hz), 153.2, 152.2, 151.2, 140.3, 133.7, 128.5 (2C), 128.2 (d, *J* = 3.1 Hz), 126.8 (d, *J* = 8.2 Hz, 2C), 122.0, 120.1 (2C), 116.0 (d, *J* = 21.8 Hz, 2C), 105.0, 95.8; ^19^F NMR (565 MHz, DMSO-*d*_*6*_) δ −114.5, −114.6 (m); IR (neat, cm^–1^): 3173, 3109, 1607, 1579, 1556, 1494, 1453, 1313, 1229, 1160, 920, 831; HRMS (ASAP+, *m*/*z*): found 305.1206, calcd for C_18_H_14_N_4_F, (M+H)^+^, 305.1202.

#### 4.3.22 6-(4-Bromophenyl)-*N*-phenyl-7*H*-pyrrolo[2,3-*d*]pyrimidin-4-amine (**18**)

4-Chloro-6-(4-bromophenyl)-7*H*-pyrrolo[2,3-*d*]pyrimidine (100 mg, 0.324 mmol, 1.0 equiv) was mixed with aniline (1.1 equiv), and 2-PrOH (5 mL) and HCl (0.61 M, 0.1 equiv) were added. The reaction mixture was stirred for 22 h at 80 °C. After cooling to room temperature, the reaction mixture was suspended in sat. Na_2_CO_3_ (aq., 2 mL) and vacuum filtered. To recover more material, the filtrate was extracted with EtOAc (4 × 10 mL). The combined organic phases were dried with brine (2 × 5 mL) and anhydrous Na_2_SO_4_, followed by filtration and concentration in vacuo. Both filtrate and precipitate were combined and dried in vacuo. The crude product was immobilized on Celite and purified using silica-gel flash chromatography (CH_2_Cl_2_/acetone, 4:1, *R*_f_ = 0.31, → CH_2_Cl_2_/acetone, 1:1). This gave 103 mg (0.282 mmol, 87%) of a white solid, mp. 329–332 °C; ^1^H NMR (600 MHz, DMSO-*d*_*6*_) δ 12.35 (s, 1H), 9.43 (s, 1H), 8.31 (s, 1H), 7.91 (d, *J* = 8.0 Hz, 2H), 7.78 (d, *J* = 8.2 Hz, 2H), 7.68 (d, *J* = 8.2 Hz, 2H), 7.35 (t, *J* = 7.7 Hz, 2H), 7.25 (s, 1H), 7.03 (t, *J* = 7.3 Hz, 1H); ^13^C NMR (150 MHz, DMSO-*d*_*6*_) δ 153.3, 152.2, 151.5, 140.3, 133.4, 132.0 (2C), 130.8, 128.5 (2C), 126.7 (2C), 122.1, 120.6, 120.2 (2C), 105.0, 96.6; IR (neat, cm^–1^): 3114, 2866, 1596, 1580, 1551, 1494, 1452, 1313, 1217, 1071, 1008, 919, 770, 746; HRMS (ASAP+, *m*/*z*): found 365.0403 calcd for C_18_H_14_N_4_Br (M+H)^+^, 365.0402.

## Supporting Information Available

Additional experiments and ^1^H and ^13^C NMR spectra of new compounds (PDF)
