# Supplementary material for: Promotion of Water as Solvent in Amination of 4-Chloropyrrolopyrimidines and Related Heterocycles under Acidic Conditions
Source: ACS Omega. 2024 Mar 12;9(12):14142–52. doi: 10.1021/acsomega.3c09673 (PMC10976386; doi:10.1021/acsomega.3c09673)
Supplement: Supplementary file 1 — ao3c09673_si_001.pdf [file ao3c09673_si_001.pdf]

Supporting Information File

Promotion of water as solvent in amination of 4-chloropyrrolopyrimidines and related heterocycles under acidic conditions

Shuhei Yasuda[1], Hanne Svergja[1], Cecilie Elisabeth Olsen[1] and Bård Helge Hoff [1]*

1 Department of Chemistry, Norwegian University of Science and Technology (NTNU), Høgskoleringen 5, NO-7491 Trondheim, Norway; e-mail HS: hannsm@stud.ntnu.no, SY: shuheiy@stud.ntnu.no, CEO: cecilie.e.olsen@ntnu.no, BHH: bard.h.hoff@ntnu.no

* Correspondence: BHH: bard.h.hoff@ntnu.no; Tel.: 0047 73593973

Contents

1. Initial amination reactions in EtOH 2
2. Amination of 1 with other acids 4
3. Amination in DMF 5
4. Amination in water with aliphatic amines 6
5. Reference to pKa values 9
6. NMR spectra of new compounds 10
7. References 30

1. Initial amination reactions in EtOH

To gain better understanding of the process we started by performing reactions with 4-chloro-7*H*-pyrrolo[2,3-d]pyrimidine (**1**) and aniline (**2a**) with varying amounts of HCl (0-5 equiv.) in EtOH. The aminations were monitored by ^{1}H NMR spectroscopy for 6 h, and integration of the H-2 protons, gave the mole ratio of the starting material **1**, the product **3a** and the side-product **4**. Table S1 shows the conversion (%) after 1 h and the mole % of compounds after 6 h.

Table S1. Effect of HCl amount on reaction progress and formation **3a** and the side-product **4**.

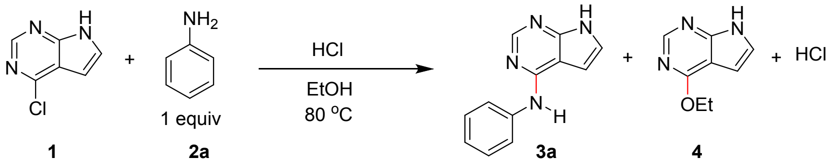

1 **2a** **3a** **4**

			Mole % after 6 h.[2]		
**Entry**	**HCl (equiv)**	**Conv. 1 h (%)** [1]	**1**	**3a**	**4**
1	0	< 1	31	69	<1
2	0.1	51	< 1	>98	< 1
3	0.5	78	< 1	90	10
4	1.0	82	< 1	86	14
5	3	82	< 1	85	15
6	5	86	< 1	83	17

[1] Conversion was measured by ^{1}H NMR, conv (%) = 100×[**3a**+**4**]/[**1**+**3a**+**4**].
[2] Mole % of **1**, **3a** and **4.** Values denoted as < 1 means not detected.

The degree of conversion after 1 h is indicative of the initial rate of the reaction. Without acid (entry 1) the product was not detected after 1 h, showing that the amination is only slowly catalyzed by EtOH hydrogen bonding. However, as small amount of **3a** and HCl is produced, the rate of this reaction is elevated. Overall, the initial rate increases with the amount of acid used, and clearly, HCl has a catalytic effect on the amination. However, at elevated levels of HCl (0.5-5 equiv., entries 3-6), the side product **4** was also produced, consuming starting

material. This is explained by excess acid deactivating the aniline by protonation, which allows EtOH to be a competitive nucleophile.

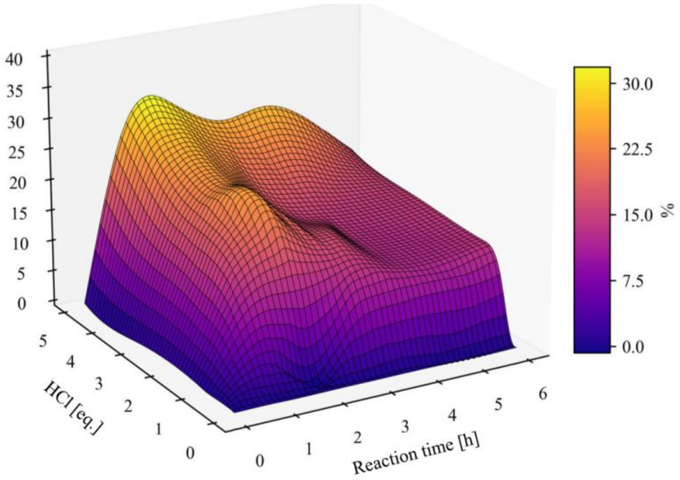

Figure S1. Effect of HCl equiv. and reaction time on level of the side-product **4**.

The levels of the side-product **4** as a function of HCl equiv. and reaction time is plotted in Figure S1. The highest concentrations were noted early in the process using 1-5 equiv. of HCl. On progression the amount of **4** decreases, showing that **4** is not just a side-product, but also a slow reacting substrate. Thus, the amount of acid should be kept low to minimize side-product formation, and in our model system 0.1 equiv. was suitable (Table S1, entry 2).

2. Amination of 1 with other acids

Depending on if the chemistry should be performed in an industrial or educational setting, the amination can also be performed with a cheap acid (H_2SO_4) or a safer acid (acetic acid). Figure S2 compares the reaction profile of aminations with HCl, H_2SO_4 and acetic acid.

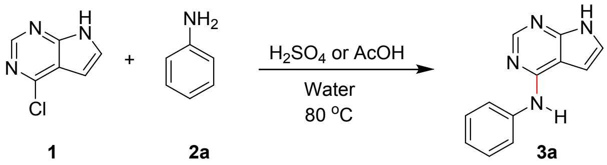

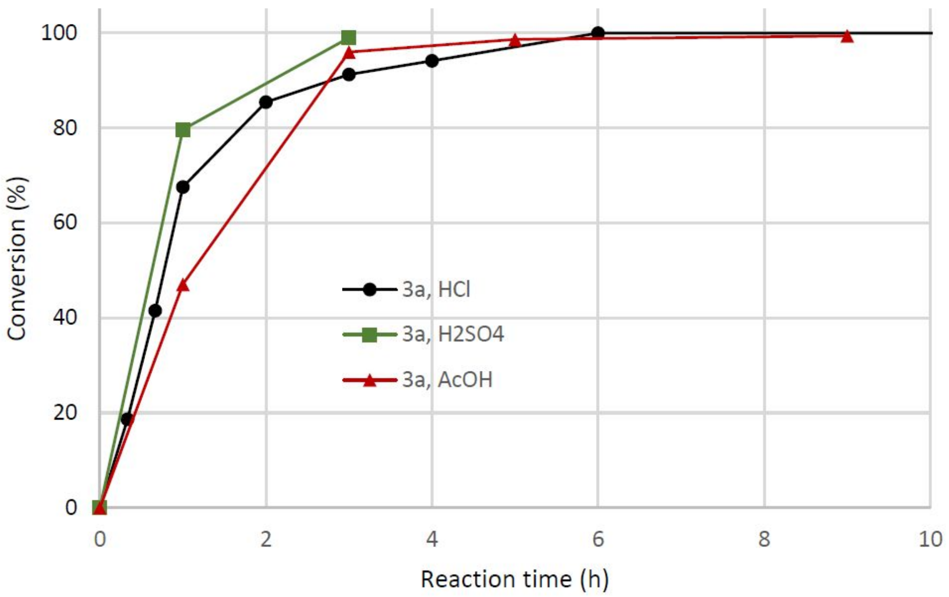

Figure S2. Comparison of the reaction profile for aminations with HCl, H_2SO_4 and acetic acid.

3. Amination in DMF

DMF is a common solvent in nucleophilic aromatic substitution. Figure S3 compares the reaction profile in DMF, DMF/water with that in water only.

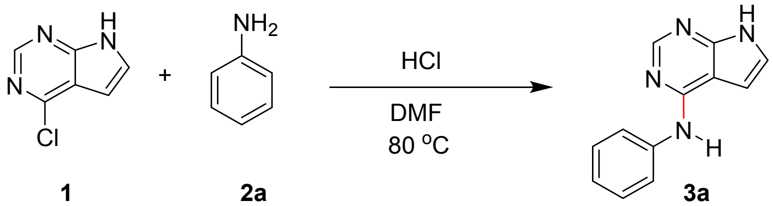

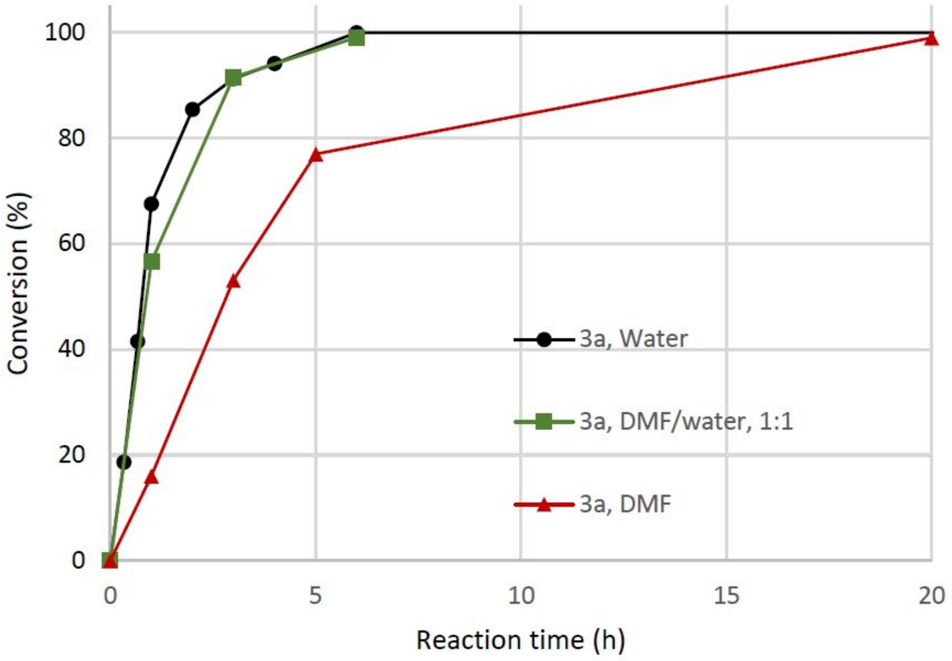

Figure S3. Comparison of reaction profile for reaction in DMF, DMF/water (1/1 by vol %) with pure water at 80 °C with 0.1 equiv of HCl.

4. Amination in water with aliphatic amines

Highly basic amines should be unsuited for amination under acidic conditions as they would be protonated by the acid catalyst. Thus, for comparison with the aniline aminations we also performed a few experiments under acidic conditions with 2,2,2-trifluoro-1-phenylethylamine (pKa = 6.1 [1]) morpholine (pKa = 8.33 [2]), 1-phenylethylamine (pKa = 9.45 [2]) and cyclohexylmethanamine (pKa = 10.49 [3]). The results are summarized in Table S2, entries 1-4.

Table S2. Amination of non-aromatic amines under different conditions.

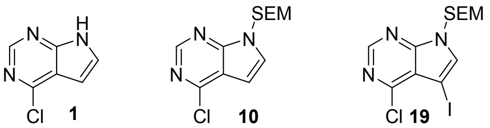

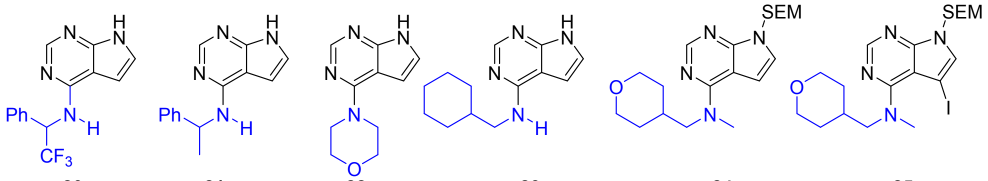

20 21 22 23 24 25

Entry	Substrate	Amine (equiv.)	HCl (equiv.)	Solvent	Reaction time (h)	Conv. (%)	Yield	Product
1	**1**	1.1	0.1	H_2O	22	low	**ND**	**20** [1]
2	**1**	1.1	0.1	H_2O	22	13	**ND**	**21**[2]
3	**1**	1.1	0.1	H_2O	22	61	**ND**	**22**
4	**1**	1.1	0.1	H_2O	22	24	**ND**	**23**
5[3)]	**1**	3	1.0	2-PrOH	6	>98	**ND**	**22**
6	**1**	3	None	H_2O	2.5	>98	**88**	**22**
7	**1**	1.5	None	H_2O	22	86	**ND**	**22**
8[2)]	**1**	3	1.0	2-PrOH	22	93	**ND**	**23**
9	**1**	3	None	H_2O	8	>98	**92**	**23**
10	**10**	3	None	H_2O	6	>98	**86**	**24**
11[3)]	**19**	3	None	H_2O	22	>98	**74**	**25**

[1] The identity of the product could not be confirmed.
[2] The identity **21** was confirmed by in-house made material.[4]
[3] Testing of the experiment reported previously [5]: HCl 3 drops corresponds to approximately 1 equiv. of HCl in this setting.
[4] The reaction temperature was 90 °C. Compound **19** was prepared as described by Reiersølmoen *et al.* [6]

Reaction with 2,2,2-trifluoroethyl-1-phenylamine in water and 0.1 equiv. of HCl gave a low conversion after 22 h (entry 1). Multiple products were formed, however their identity was not confirmed. Possibly, halogen exchange is occurring alongside amination,[7] and this amine is

concluded to be an unsuited reactant in acid catalyzed amination. 1-Phenylethylamine (entry 2) and cyclohexylmethanamine (entry 4) were as expected poor nucleophiles under these conditions. The reaction with morpholine reached a higher 61% conversion (entry 3), which is due to its lower basicity.

The morpholine and cyclohexylmethylamine derivatives **22** and **23** have previously been synthesized by Jesumoroti *et al.* [5] 4-Chloro-7*H*-pyrrolo[2,3-*d*]pyrimidine (**1**) was aminated in 2-PrOH using 3 equiv. of amine and conc. HCl (3 drops). We repeated these experiment, and weighting of conc. HCl indicated that the 3 drops corresponded to ca 1 equiv. of HCl in this case, which in essence means the reaction is run under basic conditions. The aminations worked (entries 5 and 8), but proceeded with higher rate if the acid was omitted, and the reactions are conducted in water (entries 6-7 and 9). Thus, acid should not be used in aromatic substitutions with aliphatic and benzylic amines. However, water can be a suitable solvent in basic amination of pyrrolopyrimidine, and the products **22** and **23** were isolated in 88 and 92% yield. The more lipophilic pyrrolopyrimidines **10** and **19** could also be aminated in water with *N*-methyl-1-(tetrahydro-2H-pyran-4-yl)methanamine giving **24** and **25** in yields of 86% and 74%, respectively. Due to low solubility, of **19**, a reaction temperature of 90 °C was used.

4-(7*H*-Pyrrolo[2,3-*d*]pyrimidin-4-yl)morpholine (22)

4-Chloro-7*H*-pyrrolo[2,3-*d*]pyrimidine (500 mg, 3.26 mmol, 1.0 equiv.) and morpholine (3 equiv.) were mixed with H_2O (25 mL). The reaction mixture was stirred at 80 °C for 2.5 h. After cooling to room temperature, the reaction mixture was suspended in sat. Na_2CO_3 (aq. 10 mL) and a solid formed, which was isolated by filtration. The filtrate was extracted with EtOAc (3 × 30 mL). The combined organic phases were washed with brine (30 mL), dried over anhydrous Na_2SO_4, filtered, and concentrated in vacuo. The material from the filtrate and the precipitate were combined and dried in vacuo to afford the title compound 586 mg (2.88 mmol, 88%) as an off-white solid, mp. 212 - 213 °C, (*lit.* [5] 207 °C); ^{1}H NMR (400 MHz, DMSO-d_6) δ 11.73 (br s, 1H), 8.16 (s, 1H), 7.20 (dd, J = 3.6, 2.4 Hz, 1H), 6.62 (dd, J = 3.6, 1.8 Hz, 1H), 3.84 – 3.82 (m, 4H), 3.73–3.70 (m, 4H). The ^{1}H NMR was in agreement with that reported. [5]

N-(Cyclohexylmethyl)-7*H*-pyrrolo[2,3-*d*]pyrimidin-4-amine (23)

The compound was prepared as described for preparation of **22** but using 1-cyclohexylmethanamine and reacting for 8 h. After cooling to room temperature, the reaction mixtures were suspended in sat. Na_2CO_3 (aq. 10 mL) and vacuum filtered. The filter cake was washed with water and dried in vacuo to give 690 mg (2.99 mmol, 92%) as an off-white solid, mp. 180.5 – 181.5 °C, (*lit.* [5] (179 °C); ^{1}H NMR (400 MHz, DMSO-d_6) δ 11.42 (s, 1H), 8.06 (s, 1H), 7.34 (t, J = 5.9 Hz, 1H), 7.03 (dd, J = 3.5, 1.7 Hz, 1H), 6.56 (dd, J = 3.5, 1.3 Hz, 1H), 3.30 (t, 5.6 Hz, 2H), 1.79 – 1.54 (m, 6H), 1.26 – 1.09 (m, 3H), 1.00 - 0.86 (m, 2H). The ^{1}H NMR was in agreement with that reported,[5] but we have reported the most down filed shift as multiplet.

N-Methyl-*N*-((tetrahydro-2*H*-pyran-4-yl)methyl)-7-((2-(trimethylsilyl)ethoxy)methyl)-7*H*-pyrrolo[2,3-*d*]pyrimidin-4-amine (24)

The synthesis was performed as described for compound **22** without HCl, but starting with 4-chloro-7-((2-(trimethylsilyl)ethoxy)methyl)-7*H*-pyrrolo[2,3-*d*]pyrimidine (**10**, 100 mg, 0.352 mmol). The reaction time was 6 h. Purification by silica-gel column chromatography (*n*-pentane/EtOAc, 4:1→ 1:1, R_f= 0.36) gave 114 mg (0.303 mmol, 86%) of a clear oil. ^{1}H NMR (400 MHz, DMSO-d_6) δ 8.15 (s, 1H), 7.30 (d, *J* = 3.7 Hz, 1H), 6.65 (d, *J* = 3.7 Hz, 1H), 5.50 (s, 2H), 3.87 – 3.78 (m, 2H), 3.65 (d, *J* = 7.4 Hz, 2H), 3.53 – 3.45 (m, 2H), 3.31 – 3.17 (m, 5H), 2.10 – 1.96 (m, 1H), 1.55 – 1.47 (m, 2H), 1.27 (qd, *J* = 12.0, 4.4 Hz, 2H), 0.86 – 0.75 (m, 2H), -0.10 (s, 9H); ^{13}C NMR (101 MHz, DMSO-d_6) δ 156.6, 151.1, 123.9, 102.1, 72.2, 66.8 (2C), 65.3, 57.5, 55.4, 36.1, 34.3, 34.0, 31.0, 30.2 (2C), 17.14, -1.42 (3C).

5-Iodo-*N*-methyl-*N*-((tetrahydro-2*H*-pyran-4-yl)methyl)-7-((2-(trimethylsilyl)ethoxy)methyl)-7*H*-pyrrolo[2,3-*d*]pyrimidin-4-amine (25)

The synthesis was performed as described for compound **22** without HCl, but starting with 4-chloro-5-iodo-7-((2-(trimethylsilyl)ethoxy)methyl)-7*H*-pyrrolo[2,3-*d*]pyrimidine (**19**, 100 mg, 0.244 mmol) and *N*-methyl-1-(tetrahydro-2*H*-pyran-4-yl)methanamine (3 equiv.). The reaction time was 22 h at 90 °C. Purification by silica-gel column chromatography (*n*-pentane/EtOAc, 4:1→ 1:1, R_f= 0.40) gave 91 mg (0.181 mmol, 74%) of a pale-yellow oil. ^{1}H NMR (600 MHz, DMSO-d_6) δ 8.24 (s, 1H), 7.72 (s, 1H), 5.49 (s, 2H), 3.78 – 3.75 (m, 2H), 3.56 (d, *J* = 7.4 Hz, 2H), 3.53 – 3.47 (m, 2H), 3.27 – 3.20 (m, 5H), 2.05 – 1.98 (m, 1H), 1.53 – 1.50 (m, 2H), 1.08 – 1.01 (m, 2H), 0.83 – 0.76 (m, 2H), -0.11 (s, 9H); ^{13}C NMR (151 MHz, DMSO-d_6) δ 160.1, 152.2, 150.5, 132.2, 106.0, 72.2, 66.6 (2C), 65.6, 58.5, 53.2, 42.7, 33.0, 30.4 (2C), 17.1, -1.5 (3C).

5. Reference to pKa values

Table S3. Experimental, calculated ad estimated pKa values for anilines/anilinium ions.

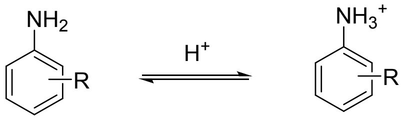

Entry	Aniline	pKa	Experimental value	Calculated/other sources
1	4-OEt (**2b**)	5.19	Not found	Estimated with Hammet constant [8] (4-hexyloxyaniline calculated to 5.59[9])
2	4-Bu (**2c**)	4.95	Not found	Estimated with Hammet constant [8] (4-Ethylaniline calculated to 5.0 [9])
3	3,4-methylene-dioxy (**2d**)	4.46	Not found	Estimated with Hammet constant [8]
4	4-F (**2e**)	4.65	From Gross et al. [8]	
5	H (**2a**)	4.58	From Gross et al. [8]	
6	3-OBn (**2f**)	ca 4.2	Not found	Hammet constant not found, Approximated from 3-methoxyaniline (pKa 4.20[8])
7	3-ethyne (**2g**)	3.82	Not found	Estimated with Hammet constant [8]
8	3-Cl (**2h**)	3.34	From Gross et al. [8]	
9	4-Br-3-F (**2i**)	2.73	Not found	Estimated with Hammet constant [8]
10	4-NO_2 (**2j**)	1.02	From Gross et al. [8]	
11	N-Me-4-F (**2k**)	ca 4.9	Not found	Approximated from *N*-methylamiline (pKa 4.89[10])
12	2-OH (2**l**)	4.84	From Tehan et al.[11]	Calculated: 4.52 [11]
13	2,6-(i-Pr)$_2$ (**2m**)	4.4	Not found	Calculated value.[9]
14	2-I (**2n**)	2.60	From Tehan et al.[11]	Calculated: 3.11 [11]
15	2,4-Cl (**2o**)	2.0	From Tehan et al.[11]	Calculated: 3.11 [11]
16	2,4,5-Cl (**2p**)	1.09	From Tehan et al.[11]	Calculated: 2.02 [11]
17	2,6-Cl (**2q**)	0.42	From Tehan et al.[11]	Calculated: 3.44 [11]
18	2-NO_2 (**2r**)	-0.31	From Eastes et al.[10]	
19	2-CF_3, 4-NO_2 (**2s**)	< 0	Not found	Estimated based on pKa of **2r**
20	2,3,4,5,6-F (**2t**)	-0.28	From Tehan et al.[11]	Calculated: -0.49 [11]

6. NMR spectra of new compounds

_N_-(4-Butylphenyl)-7_H_-pyrrolo[2,3-_d_]pyrimidin-4-amine (3c)

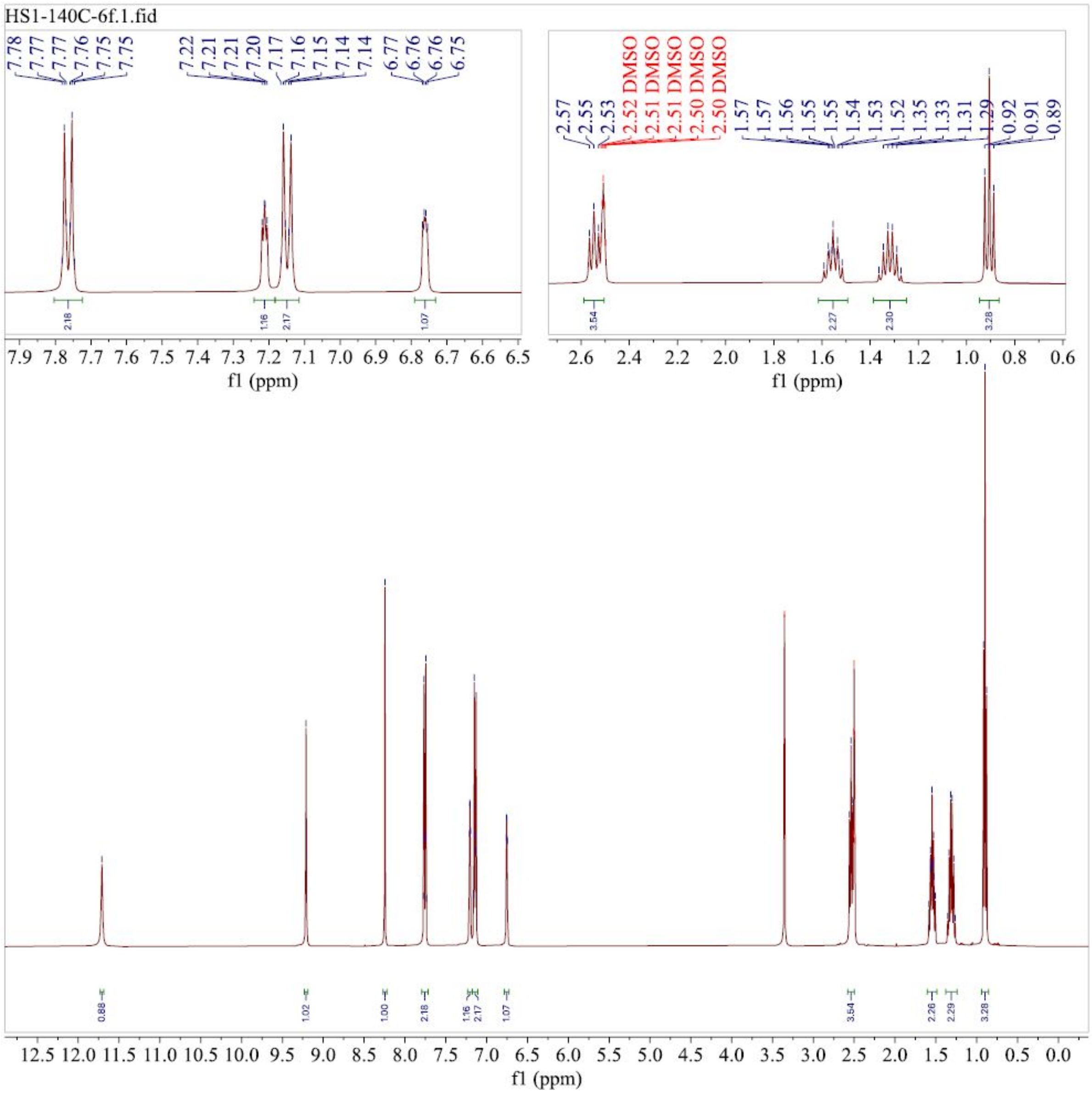

Figure S4. ^{1}H NMR (400 MHz, DMSO-d_6) of compound **3c.**

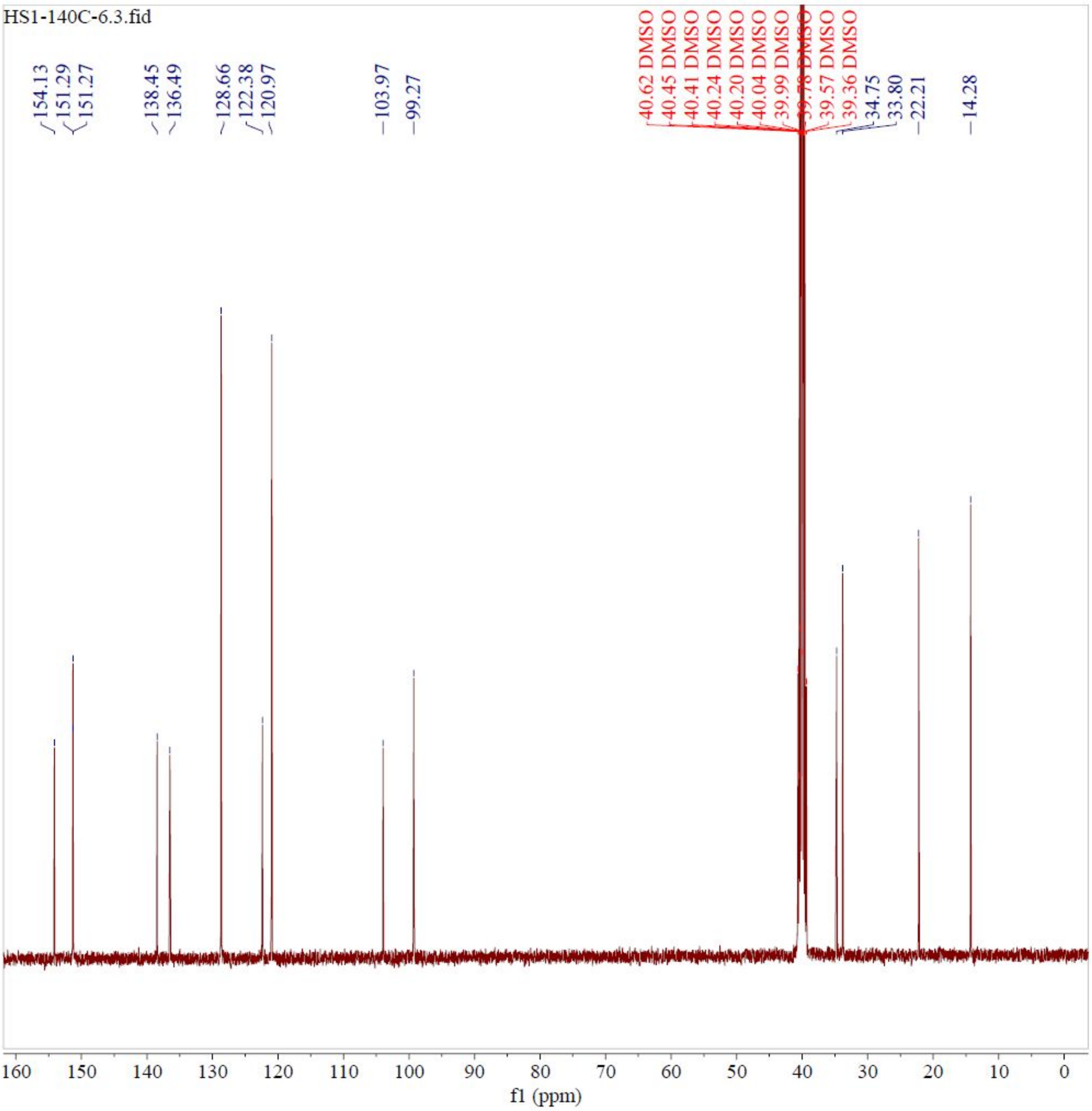

Figure S5. ^{13}C NMR (100 MHz, DMSO-d_6) of compound **3c.**

***N*-(3-(Benzyloxy)phenyl)-7*H*-pyrrolo[2,3-*d*]pyrimidin-4-amine (3f)**

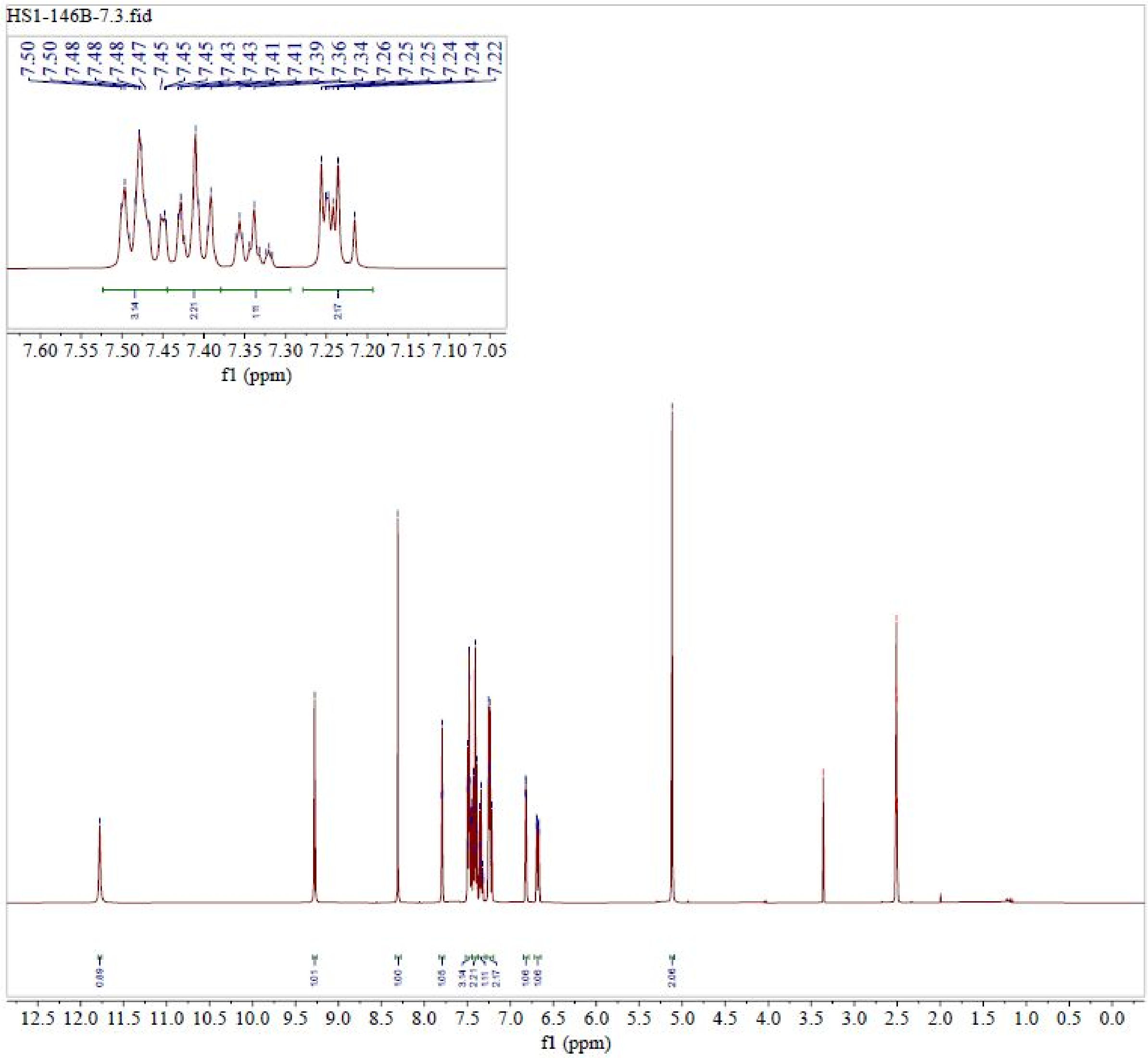

Figure S6. ^{1}H NMR (400 MHz, DMSO-d_6) of compound **3f.**

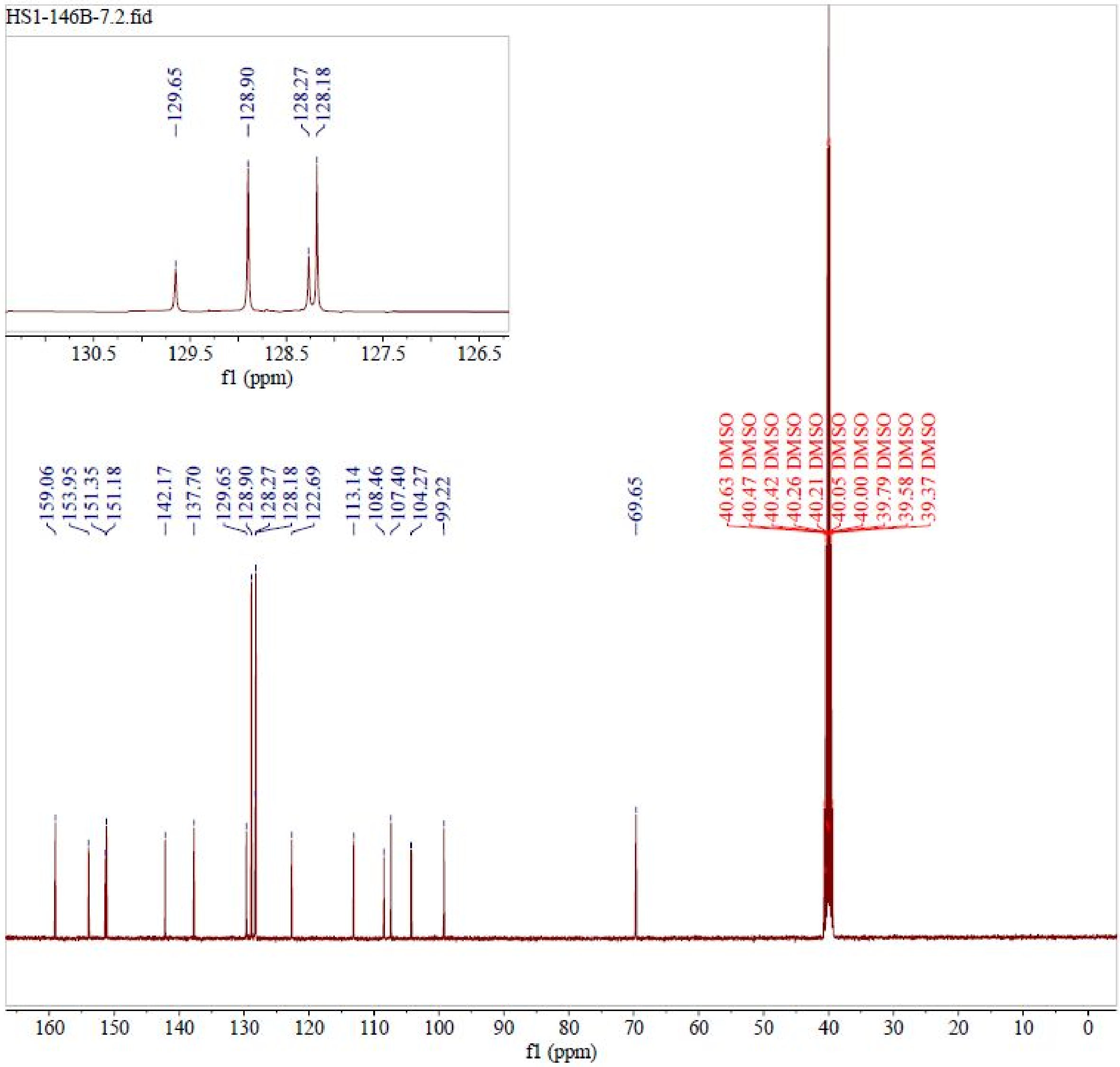

Figure S7. ^{13}C NMR (100 MHz, DMSO-d_6) of compound **3f.**

N-(3-Ethynylphenyl)-7H-pyrrolo[2,3-d]pyrimidin-4-amine (3g)

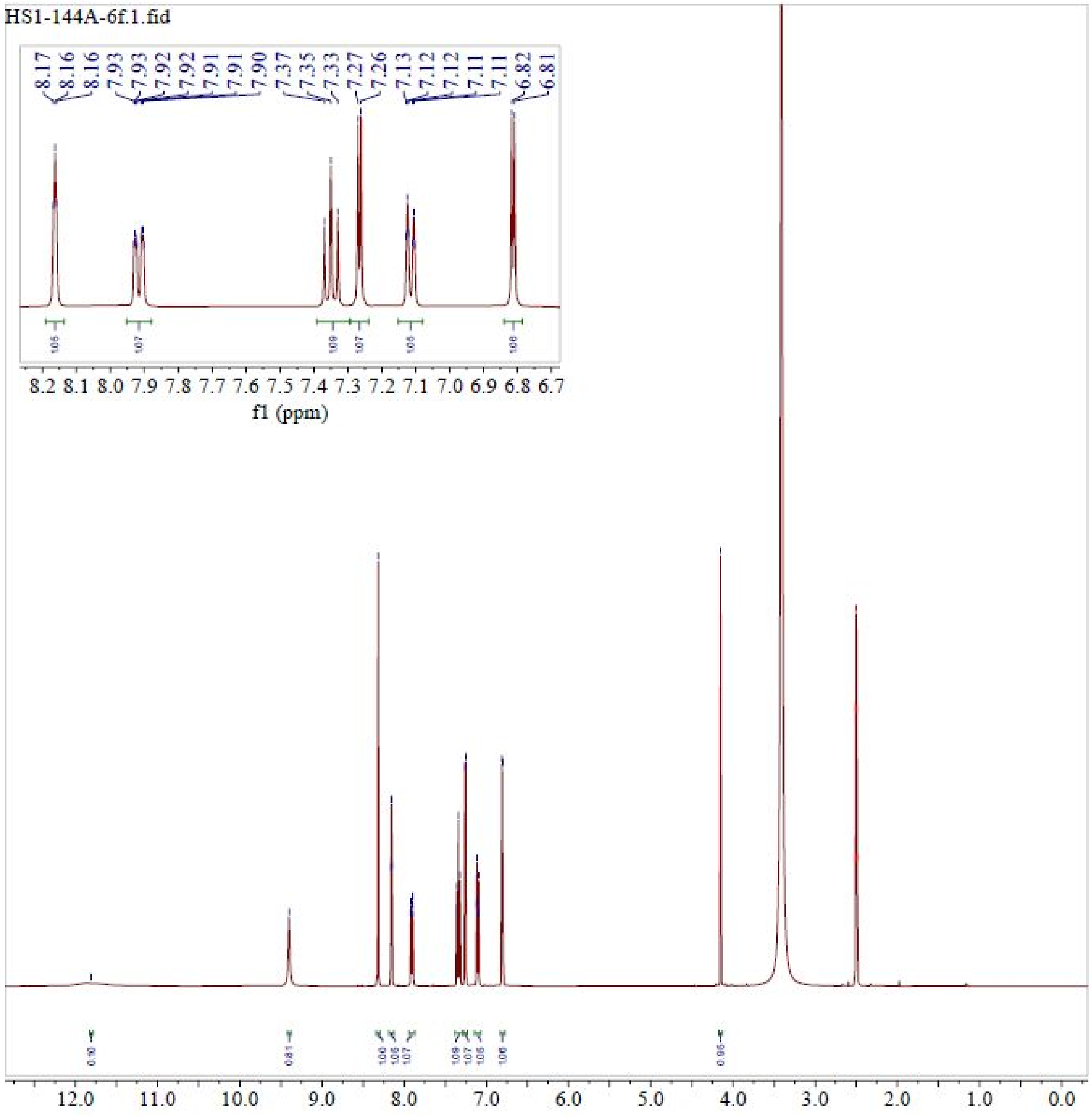

Figure S8. ^{1}H NMR (400 MHz, DMSO-d_6) of compound **3g.**

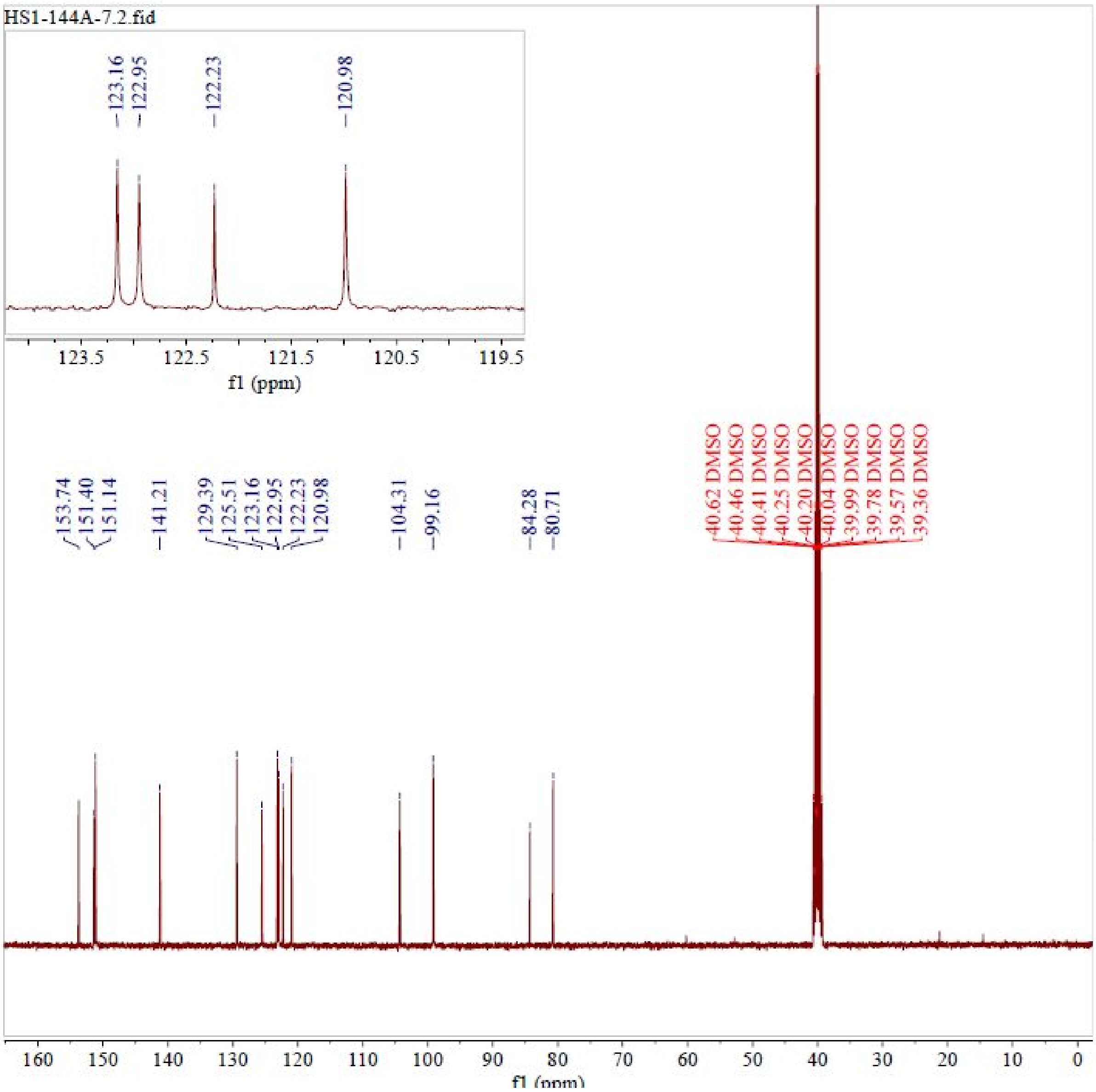

Figure S9. ^{13}C NMR (100 MHz, DMSO-d_6) of compound **3g.**

***N*-(4-Bromo-3-fluorophenyl)-7*H*-pyrrolo[2,3-*d*]pyrimidin-4-amine (3i)**

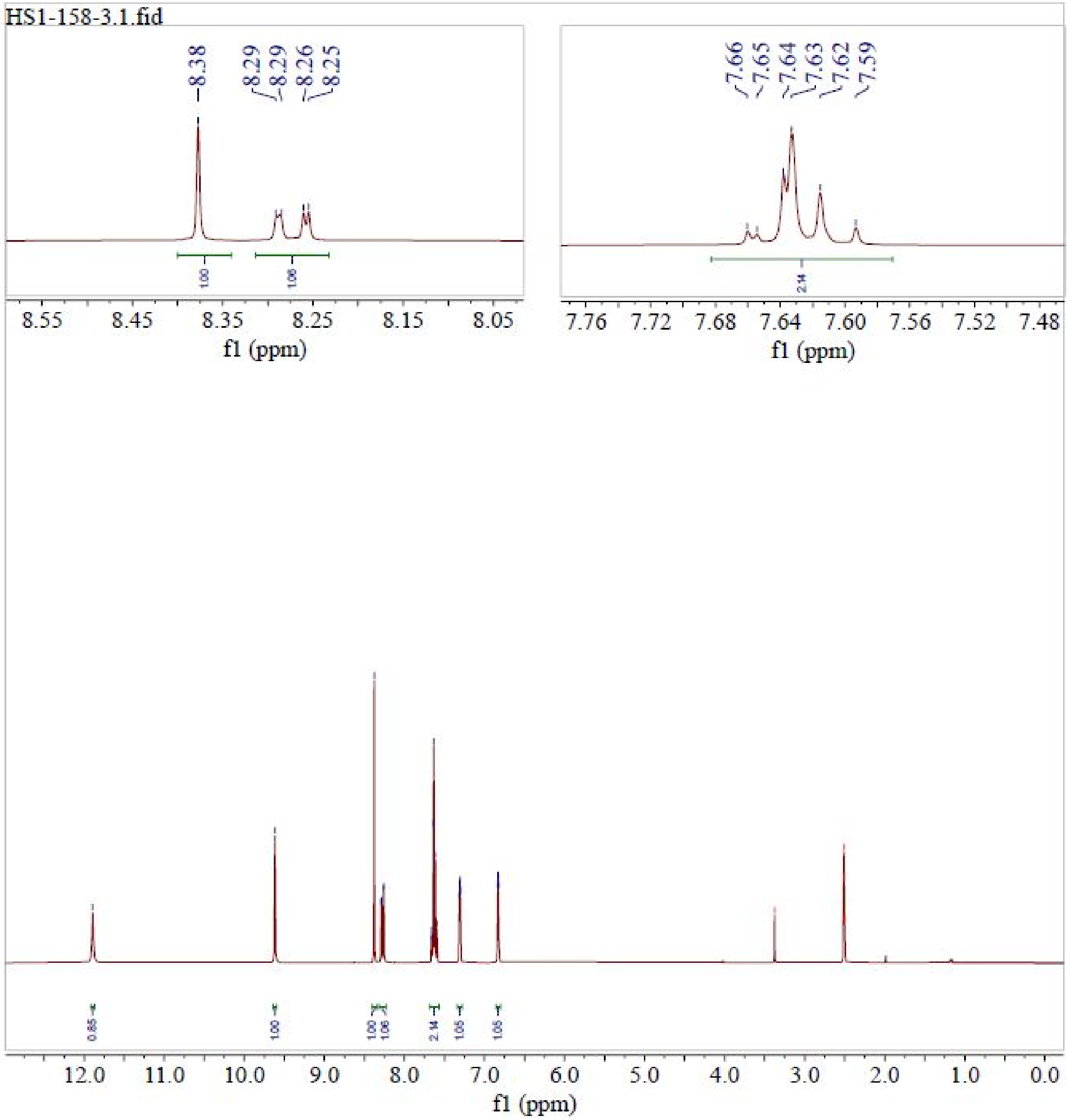

Figure S10. ^{1}H NMR (400 MHz, DMSO-d_6) of compound **3i.**

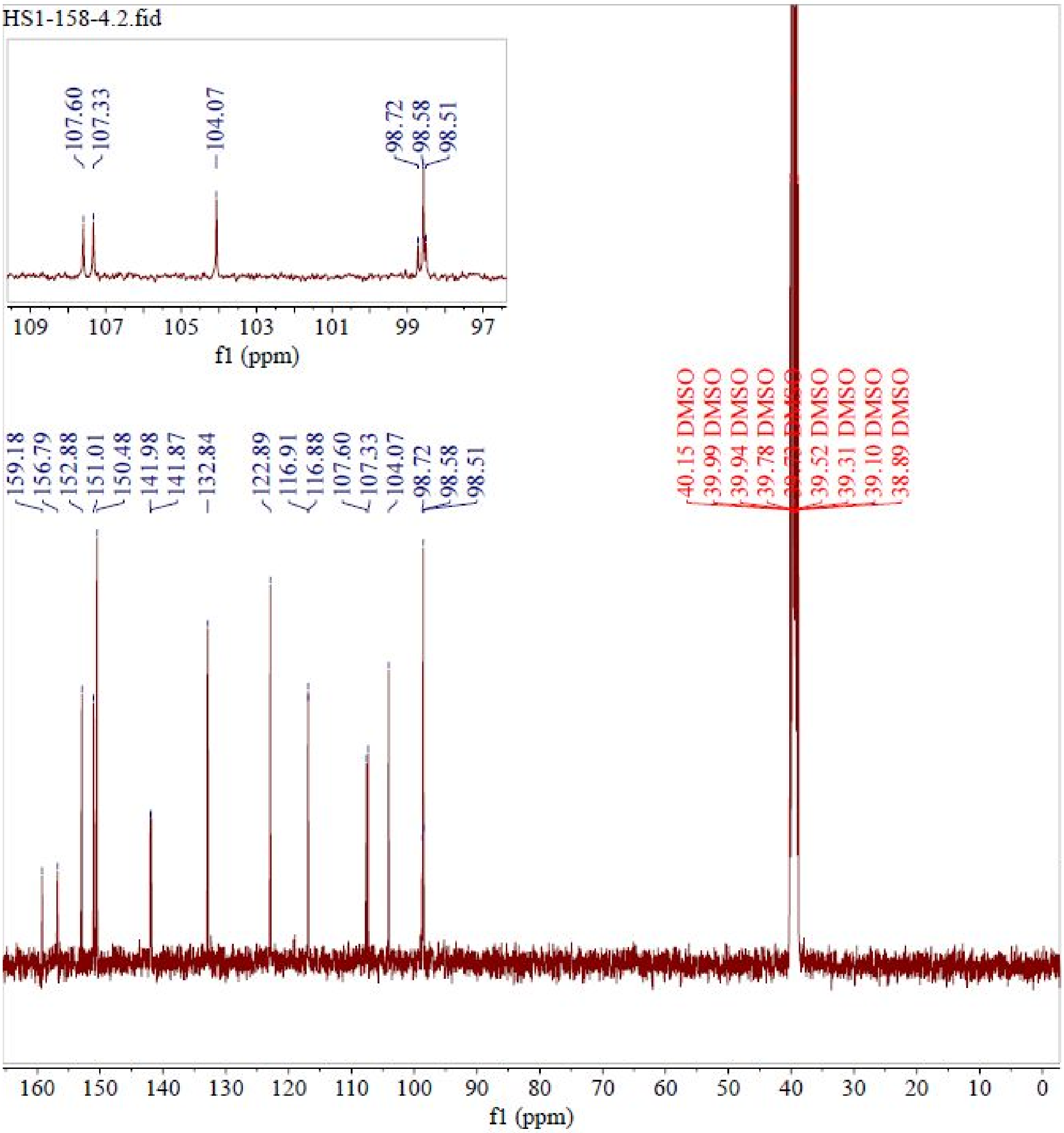

Figure S11. ^{13}C NMR (100 MHz, DMSO-d_6) of compound **3i.**

N-(4-Fluorophenyl)-*N*-methyl-7*H*-pyrrolo[2,3-*d*]pyrimidin-4-amine (3k)

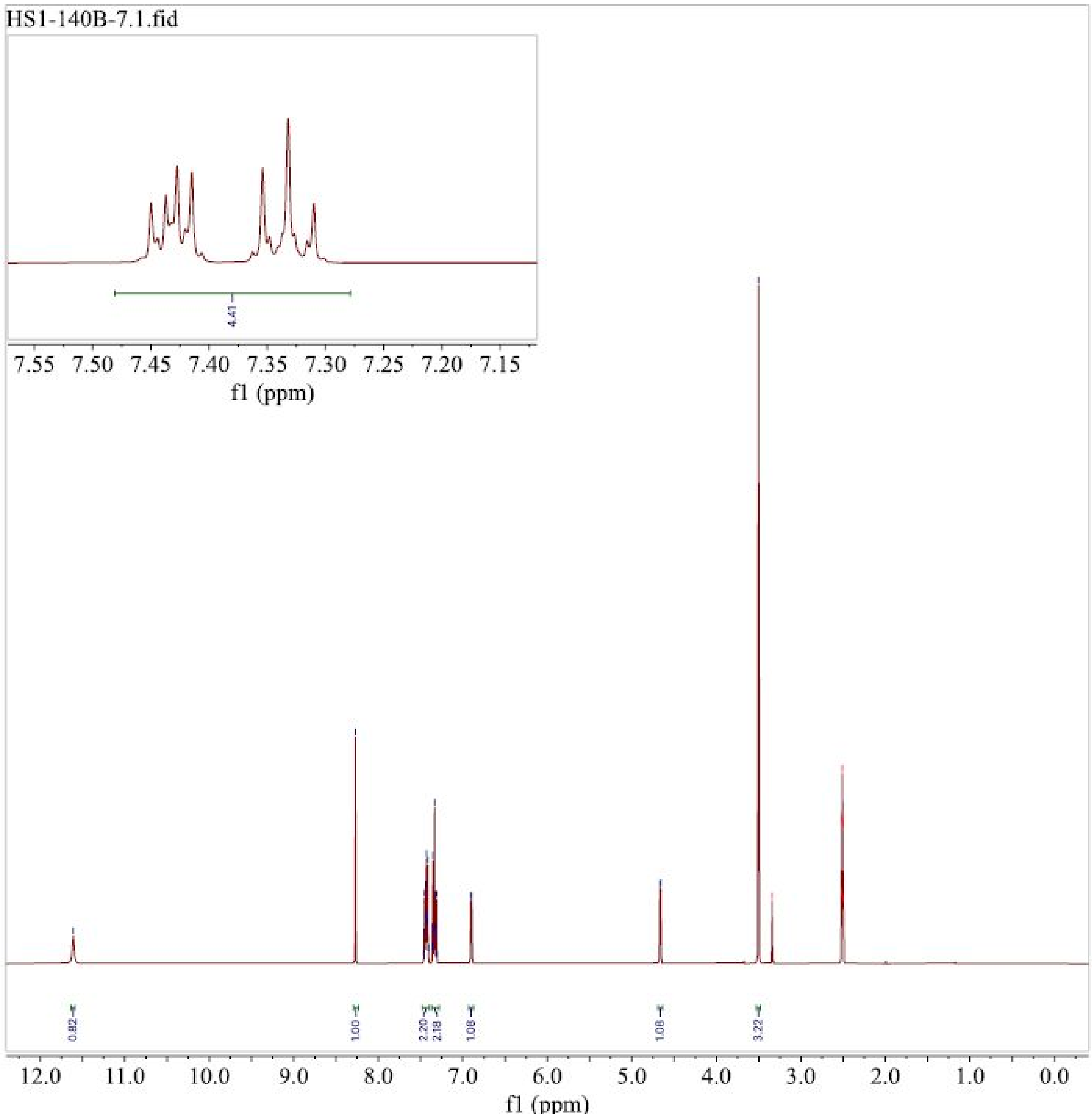

Figure S12. ^{1}H NMR (400 MHz, DMSO-d_6) of compound **3k.**

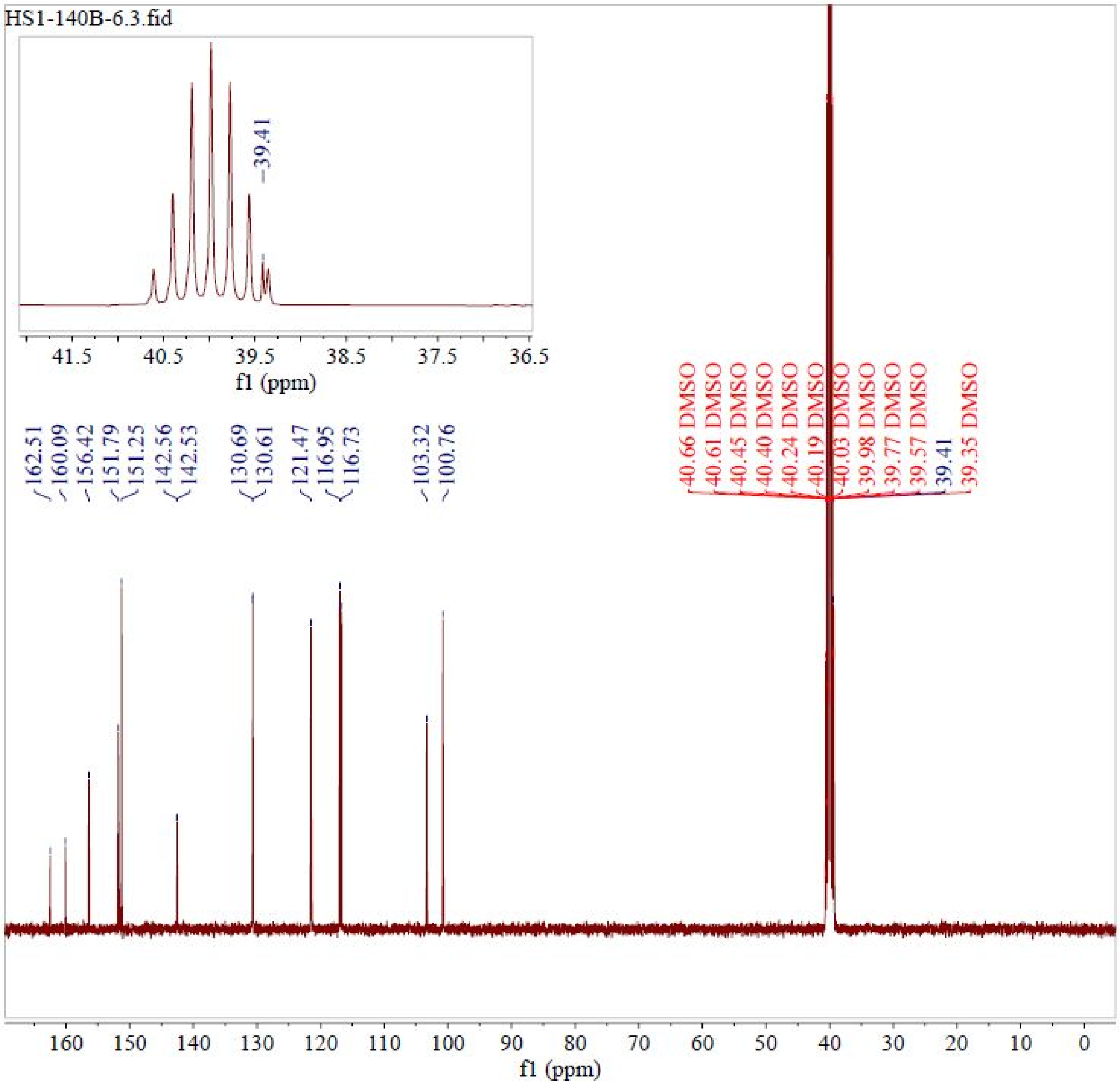

Figure S13. ^{13}C NMR (100 MHz, DMSO-d_6) of compound **3k.**

***N*-(2-Iodophenyl)-7*H*-pyrrolo[2,3-*d*]pyrimidin-4-amin (3n)**

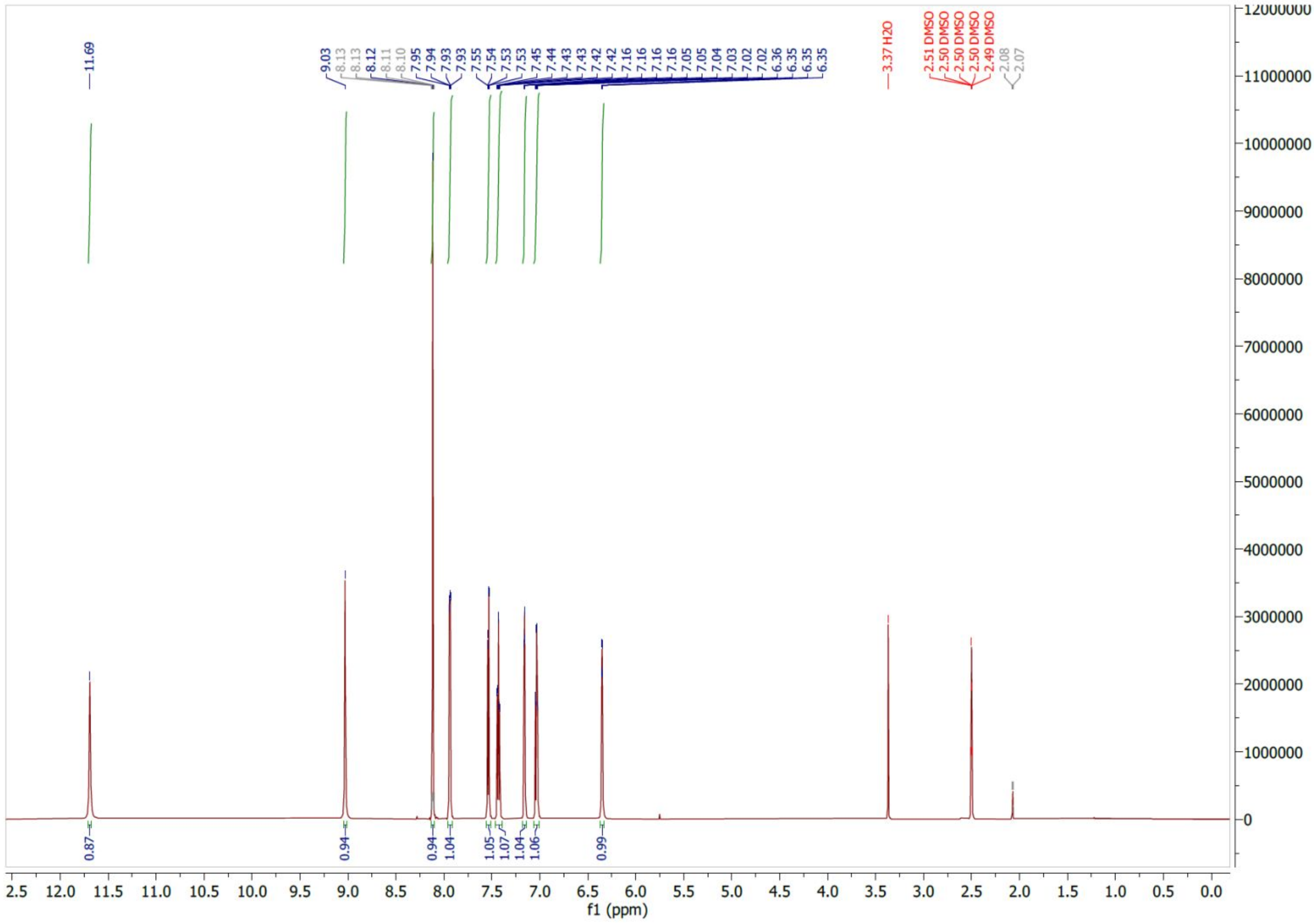

Figure S14. ^{1}H NMR (600 MHz, DMSO-d_6) of compound **3n.**

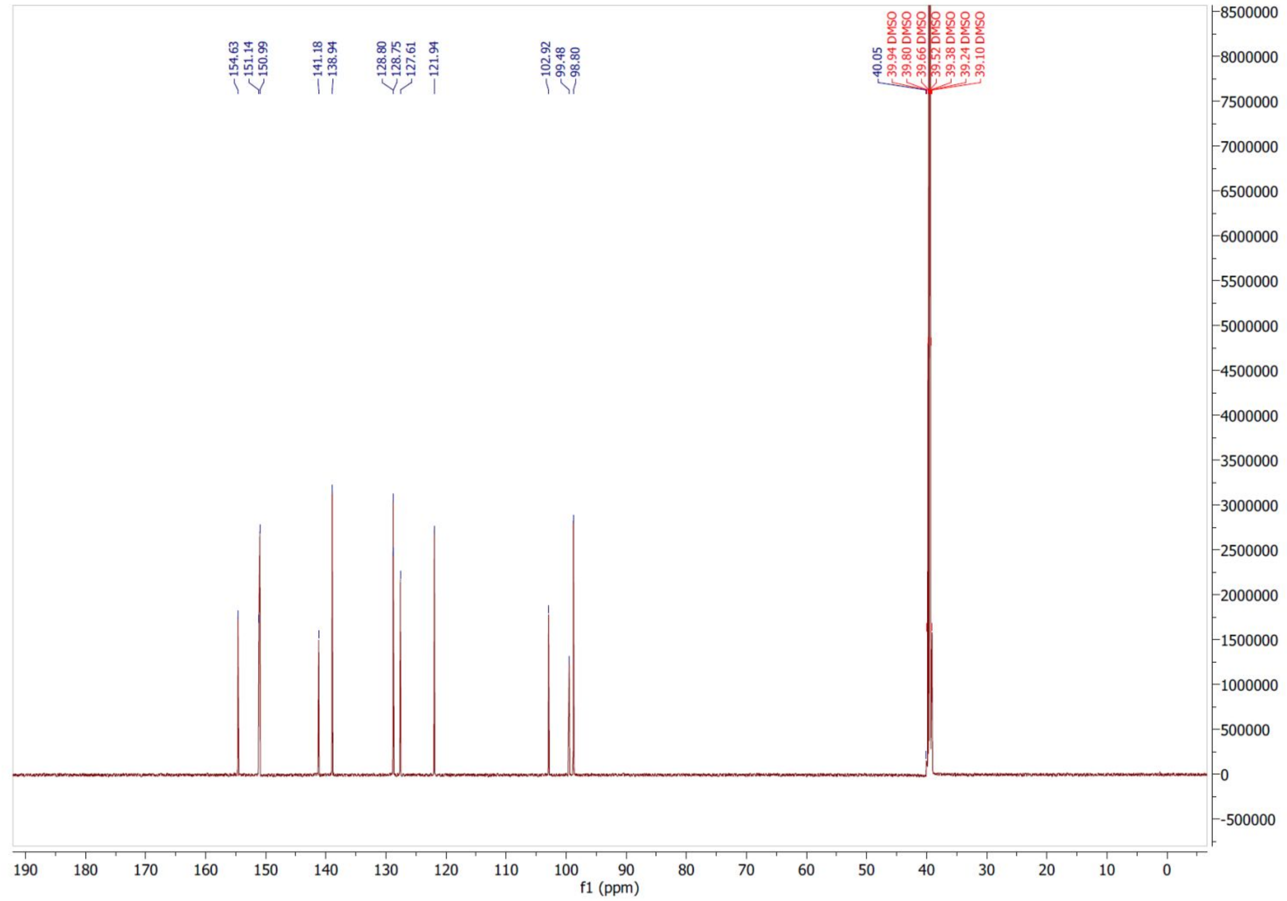

Figure S15. ^{13}C NMR (150 MHz, DMSO-d_6) of compound **3n.**

N-(2,4-Dichlorophenyl)-7H-pyrrolo[2,3-d]pyrimidin-4-amine (3o)

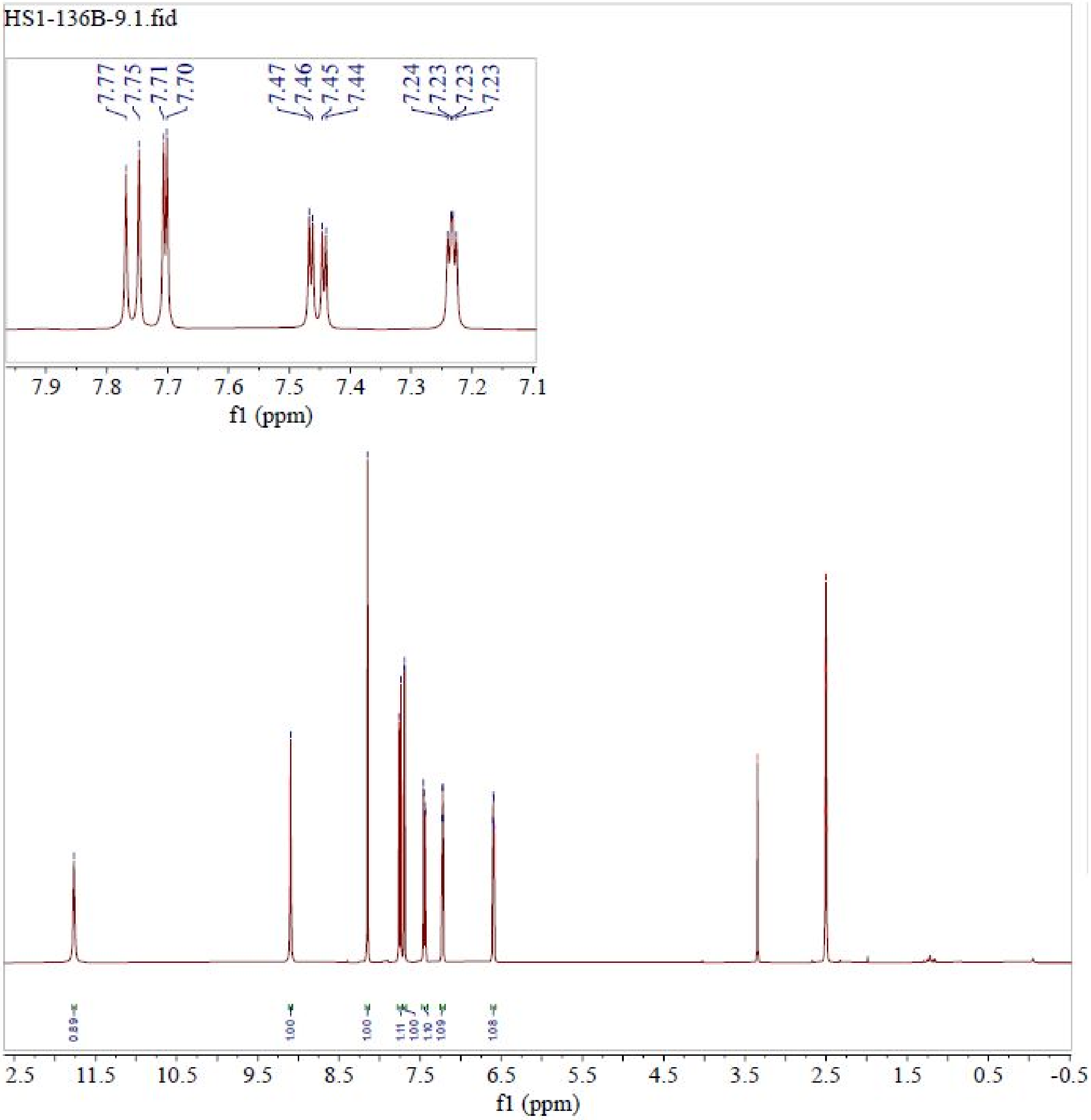

Figure S16. ^{1}H NMR (400 MHz, DMSO-d_6) of compound **3o.**

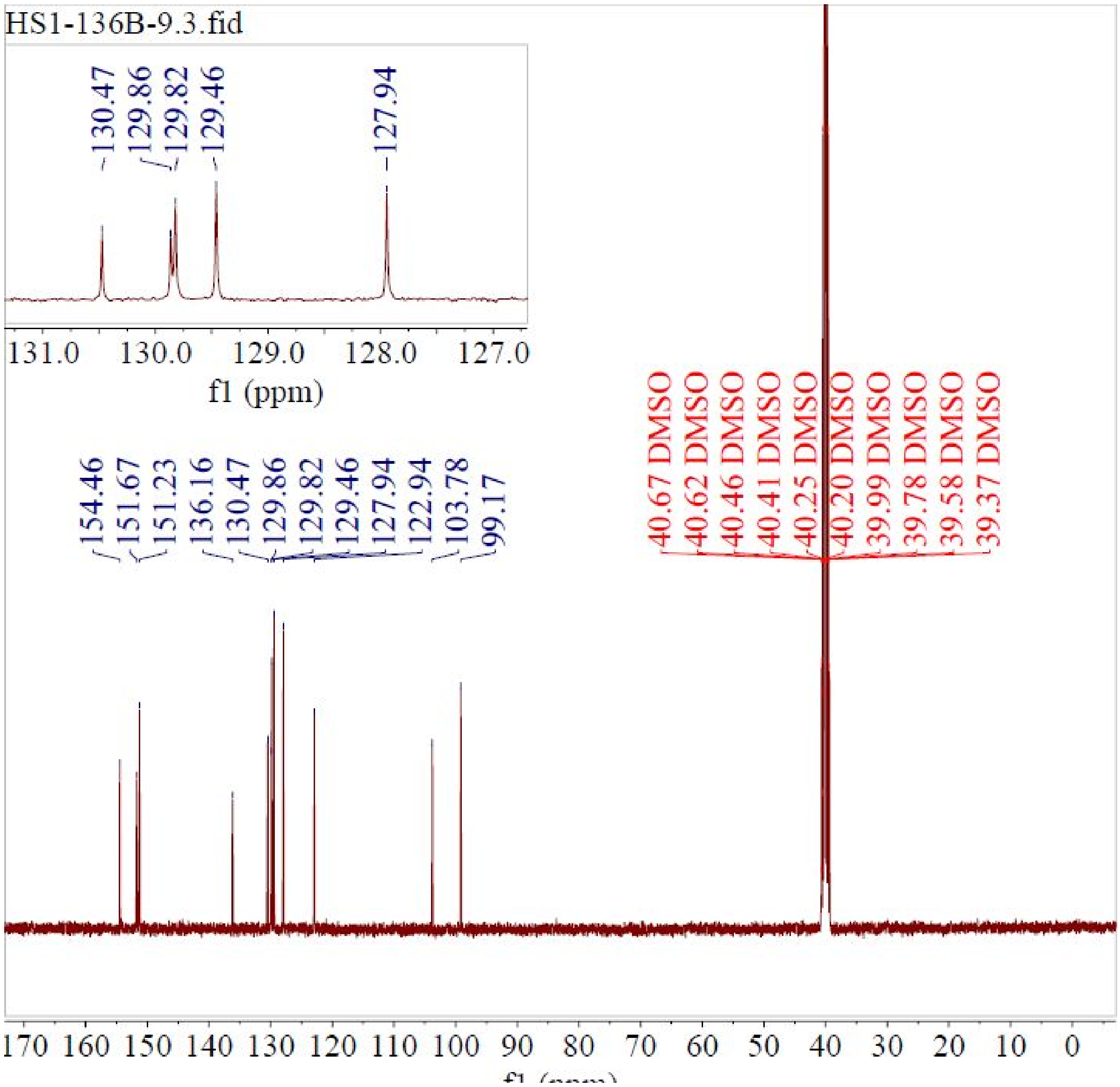

Figure S17. ^{13}C NMR (100 MHz, DMSO-d_6) of compound **3o.**

***N*-Phenyl-7-((2-(trimethylsilyl)ethoxy)methyl)-7*H*-pyrrolo[2,3-*d*]pyrimidin-4-amine (16)**

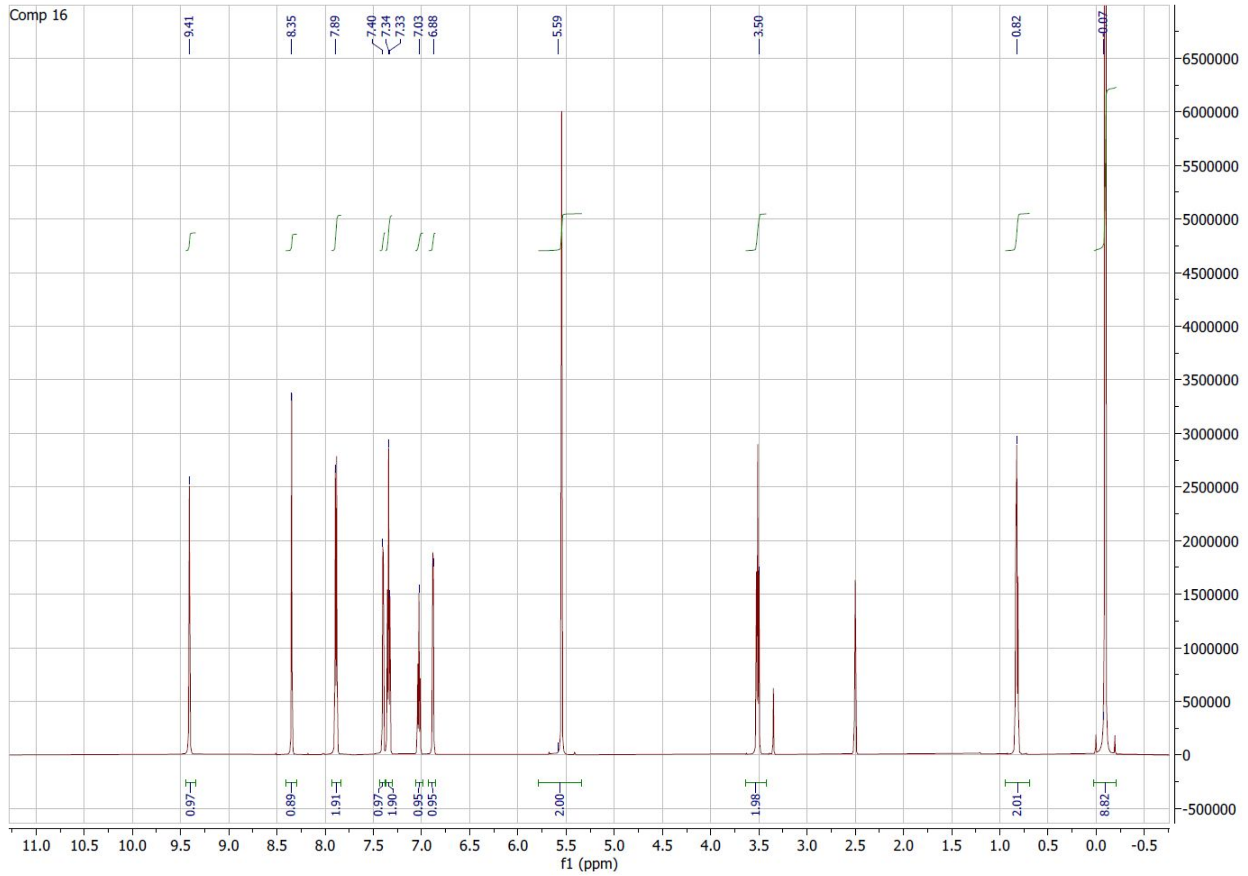

Figure S18. ^{1}H NMR (600 MHz, DMSO-d_6) of compound **16**

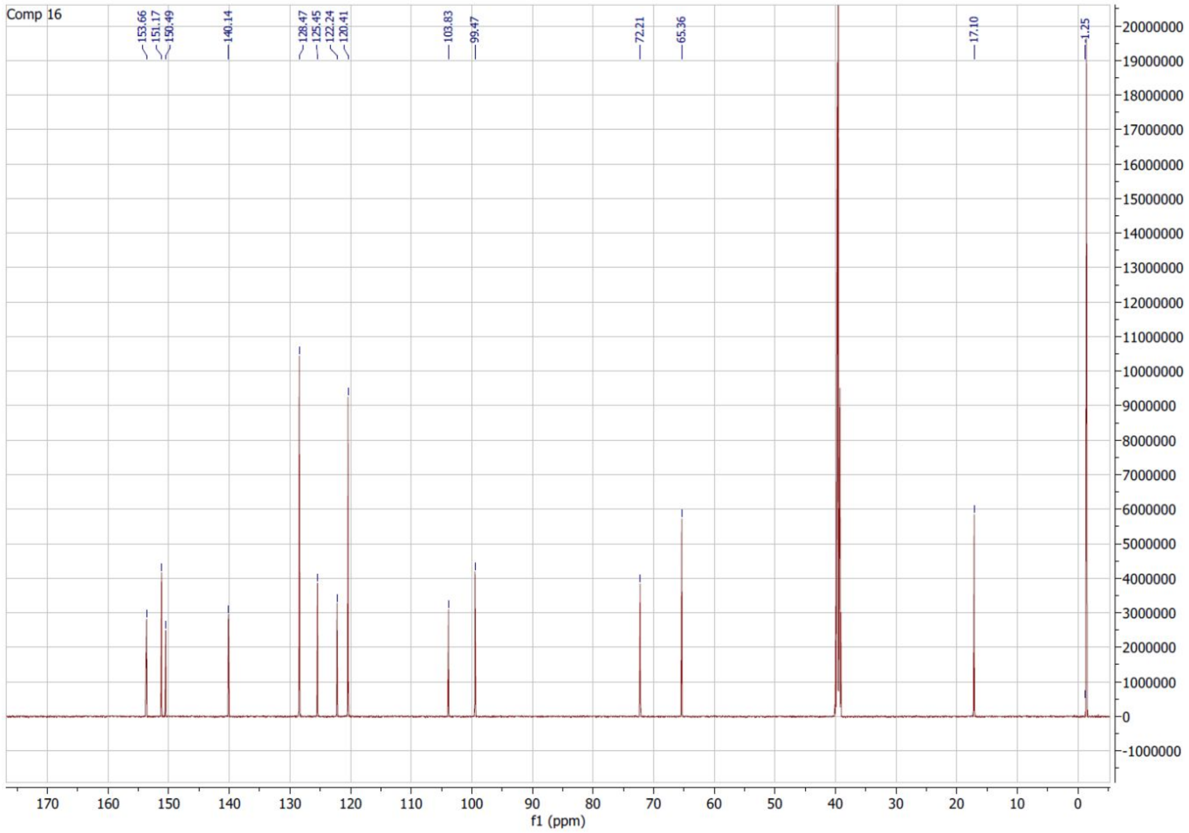

Figure S19. ^{13}C NMR (150 MHz, DMSO-d_6) of compound **16.**

6-(4-Fluorophenyl)-N-phenyl-7H-pyrrolo[2,3-d]pyrimidin-4-amine (17)

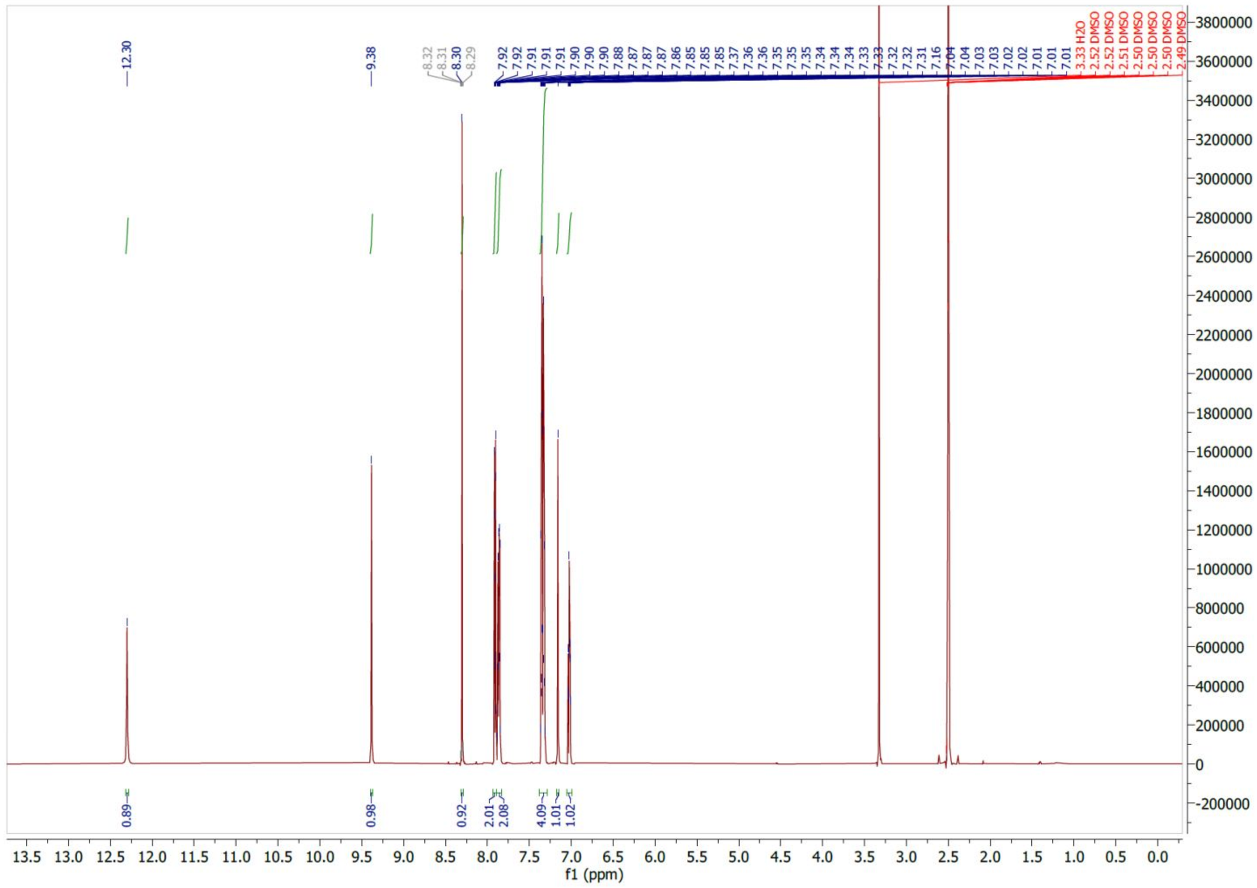

Figure S20. ^{1}H NMR (600 MHz, DMSO-d_6) of compound **17.**

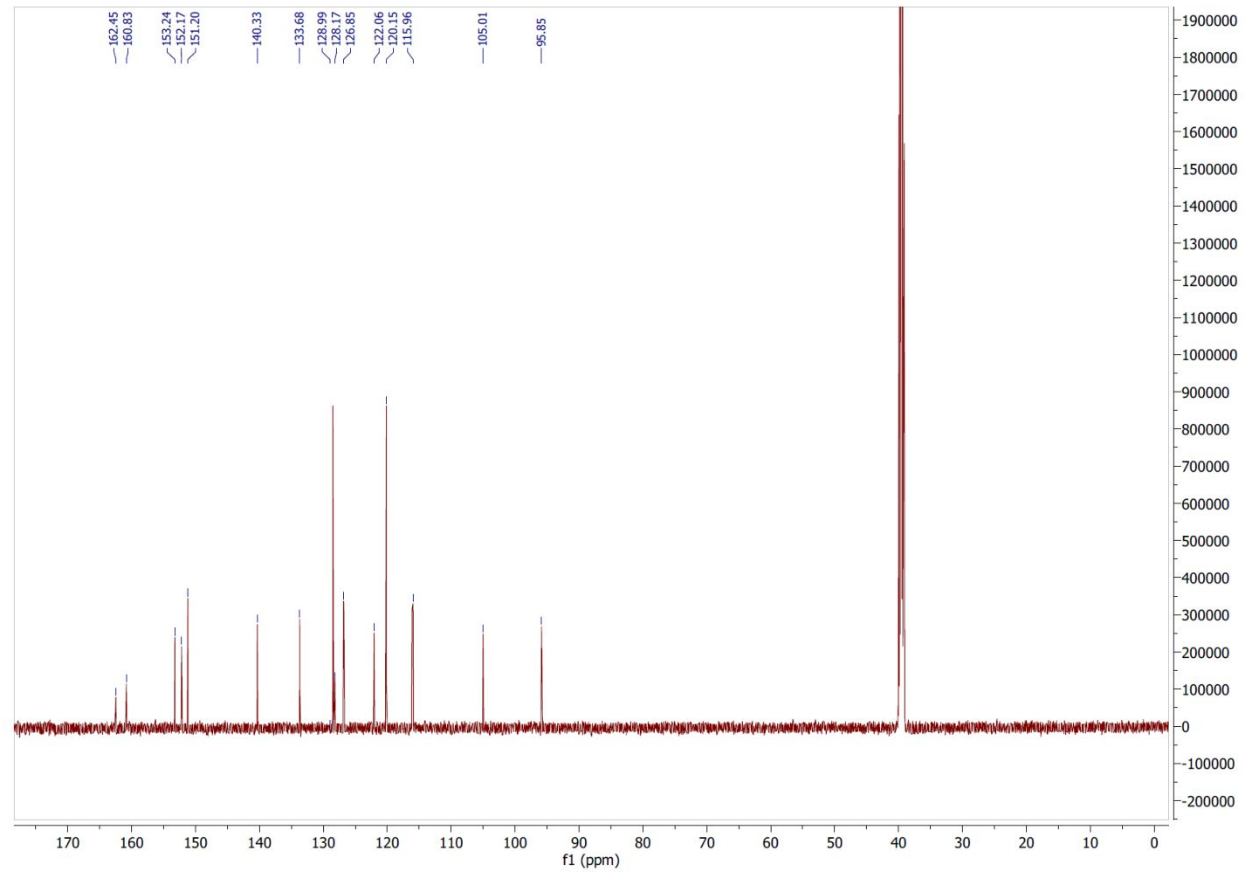

Figure S21. ^{13}C NMR (150 MHz, DMSO-d_6) of compound **17.**

6-(4-Bromophenyl)-N-phenyl-7H-pyrrolo[2,3-d]pyrimidin-4-amine (18)

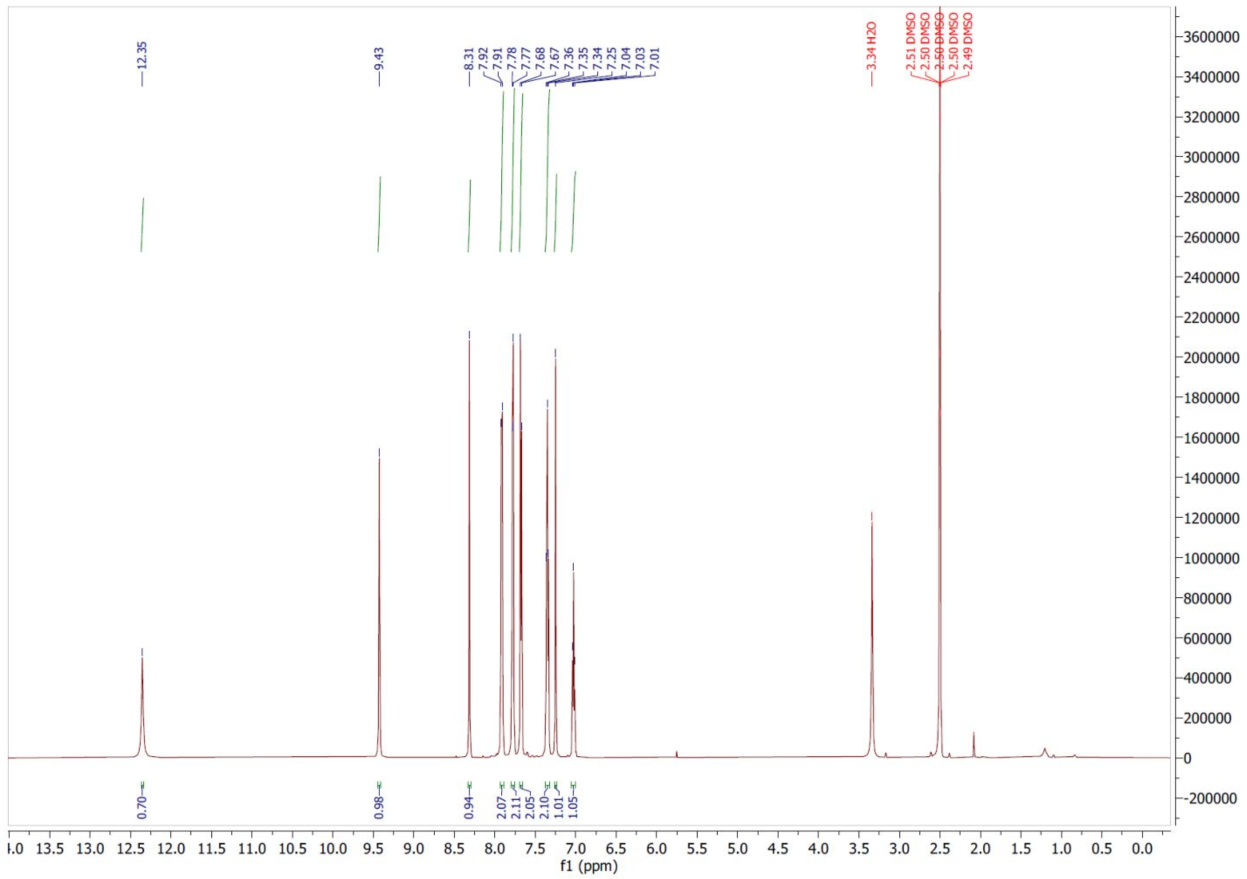

Figure S22. ^{1}H NMR (600 MHz, DMSO-d_6) of compound **18.**

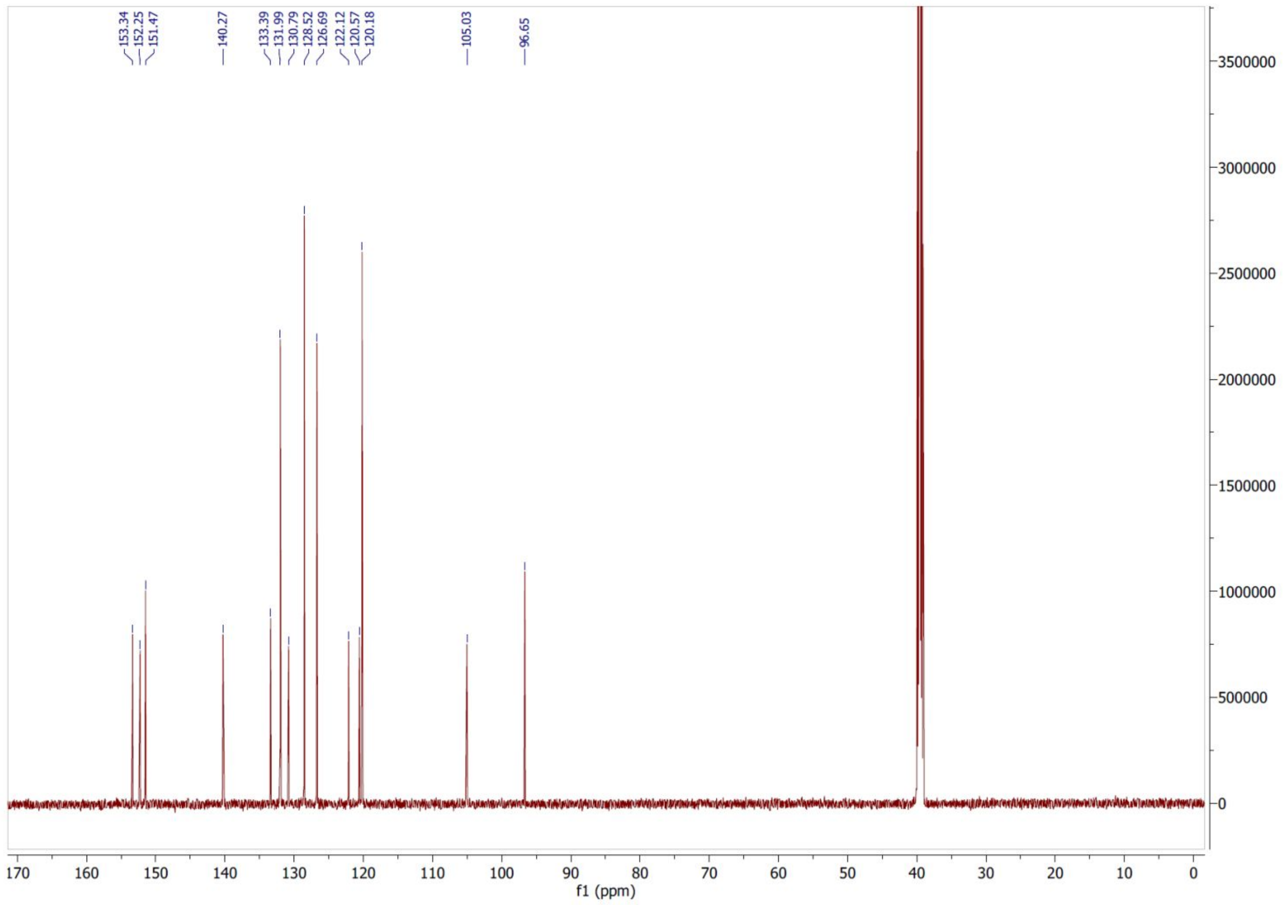

Figure S23. ^{13}C NMR (150 MHz, DMSO-d_6) of compound **18.**

7. References

(1) ChemBK. *2,2,2-Trifluoro-1-phenyl-ethylamine:* *https://www.chembk.com/en/chem/2,2,2-Trifluoro-1-phenyl-ethylamine*. 2023. (accessed 2023 05.09.2023).
(2) Smith, P. J.; Noble, A. A Primary Hydrogen-Deuterium Isotope Effect Study on the Carbonyl Elimination Reaction of 9-Fluorenyl Nitrate with Various Bases. *Can. J. Chem.* **1975**, *53*, 263-268. DOI: https://cdnsciencepub.com/doi/pdf/10.1139/v75-036.
(3) Juranic, I. Simple Method for the Estimation of pKa of Amines. *Croat. Chem. Acta* **2014**, *87*, 343-347. DOI: http://dx.doi.org/10.5562/cca2462.
(4) Aarhus, T. I.; Bjørnstad, F.; Wolowczyk, C.; Larsen, K. U.; Rognstad, L.; Leithaug, T.; Unger, A.; Habenberger, P.; Wolf, A.; Bjørkøy, G.; et al. Synthesis and Development of Highly Selective Pyrrolo[2,3-d]pyrimidine CSF1R Inhibitors Targeting the Autoinhibited Form. *J. Med. Chem.* **2023**, *66*, 6959-6980. DOI: https://doi.org/10.1021/acs.jmedchem.3c00428.
(5) Jesumoroti, O. J.; Beteck, R. M.; Jordaan, A.; Warner, D. F.; Legoabe, L. J. Exploration of 4-aminopyrrolo[2,3-d]pyrimidine as antitubercular agents. *Mol. Divers.* **2023**, *27*, 753-756. DOI: https://doi.org/10.1007/s11030-022-10453-1.
(6) Reiersølmoen, A. C.; Han, J.; Sundby, E.; Hoff, B. H. Identification of fused pyrimidines as interleukin 17 secretion inhibitors. *Eur. J. Med. Chem.* **2018**, *155*, 562-578. DOI: https://doi.org/10.1016/j.ejmech.2018.06.019.
(7) Lansbergen, B.; Meister, C. S.; McLeod, M. C. Unexpected rearrangements and a novel synthesis of 1,1-dichloro-1-alkenones from 1,1,1-trifluoroalkanones with aluminium trichloride. *Beilstein J. Org. Chem.* **2021**, *17*, 404-409. DOI: https://doi.org/10.3762/bjoc.17.36.
(8) Gross, K. C.; Seybold, P. G.; Peralta-Inga, Z.; Murray, J. S.; Politzer, P. Comparison of Quantum Chemical Parameters and Hammett Constants in Correlating pKa Values of Substituted Anilines. *J. Org. Chem.* **2001**, *66* (21), 6919-6925. DOI: https://doi.org/10.1021/jo010234g.
(9) Broderius, S. J.; Kahl, M. D.; Hoglund, M. D. Use of joint toxic response to define the primary mode of toxic action for diverse industrial organic chemicals. *Environ. Toxicol. Chemi.* **1995**, *14* (9), 1591-1605. DOI: https://doi.org/10.1002/etc.5620140920.
(10) Eastes, J. W.; Aldridge, M. H.; Kamlet, M. J. Effects of N-alkylation and N,N-dialkylation of the pKa of anilinium and nitroanilinium ions. *J. Chem. Soc. (B)* **1969**, 922-928. DOI: https://doi.org/10.1039/J29690000922.
(11) Tehan, B. G.; Lloyd, E. J.; Wong, M. G.; Pitt, W. R.; Gancia, E.; Manallack, D. T. Estimation of pKa Using Semiempirical Molecular Orbital Methods. Part 2: Application to Amines, Anilines and Various Nitrogen Containing Heterocyclic Compounds. *Quant. Struct.-Act. Relat.* **2002**, *21* (5), 473-485. DOI: https://doi.org/10.1002/1521-3838(200211)21:5<473::AID-QSAR473>3.0.CO;2-D.
